# Schizotypal personality traits and the social learning of fear

**DOI:** 10.1038/s41598-021-02336-6

**Published:** 2021-11-29

**Authors:** Antonio González-Rodríguez, Ángel García-Pérez, Marta Godoy-Giménez, Isabel Carmona, Ángeles F. Estévez, Pablo Sayans-Jiménez, Fernando Cañadas

**Affiliations:** 1 Department of Psychology, University of Almería, 04120 Almería, Spain; 2 CEINSA Health Research Centre, University of Almería, 04120 Almería, Spain; 3 CERNEP Research Centre, University of Almería, 04120 Almería, Spain

**Keywords:** Emotion, Learning and memory, Neuroscience, Physiology, Psychology

## Abstract

Schizotypy can be defined as a combination of traits qualitatively similar to those found in schizophrenia, but milder in their expression, that can be found in clinical and non-clinical populations. In this research, we explore, to our knowledge, for the first time, whether schizotypal personality traits may affect the acquisition of conditioned fear by social means only. Apart from being an essential capacity to ensure learning in safe environments, social fear learning shares important characteristics with direct fear acquisition, which also makes it a great candidate for developing successful extinction procedures. Undergraduate students (n = 72) performed a task of social fear learning. In this task, participants watched a video of a person that simulated to receive electric shocks (unconditioned stimulus; US) paired with a coloured square (conditioned stimulus plus; CS+), while another coloured square was never paired (conditioned stimulus minus; CS−) with the shock. After that, they were presented with a similar sequence of coloured screens. Their Skin Conductance Responses (SCRs) were registered during the whole process. Once they finished, they completed the Schizotypal Personality Questionnaire (SPQ). Our results revealed that participants with a low score in the Cognitive-Perceptual factor of the SPQ exhibited higher SCRs when they saw the US than when they saw the CS− (all *p*s < 0.01) during the learning phase. Nevertheless, those with higher scores did not present any difference in their SCRs toward both stimuli (all *p*s > 0.05), a pattern that has been similarly found in schizophrenia. During the final trials of the test phase, participants with the highest scores in the Disorganized factor were the only ones that maintained a higher SCR towards the CS+ than towards the CS− (*p* = 0.006), which could be associated with an impairment in their extinction processes.

## Introduction

Schizotypy may be conceptualized as a manifestation of traits similar to those presented in schizophrenia, but milder in their expression, which can be found in both clinical and non-clinical populations^1–3^ with different degrees of severity^4–6^. Literature has suggested that showing high levels of these traits may be a risk factor for developing psychosis spectrum disorders^7,8^. Throughout the present manuscript, we will refer to this set of traits that can also be present in non-clinical populations as “schizotypal personality traits”. Consistent evidence suggests that these traits are organized within three main factors^9–13^. The Cognitive-Perceptual factor, sometimes called Positive Schizotypy, is related to magical thinking, unusual perceptual experiences, ideas of reference, and paranoid ideation. The Interpersonal factor, sometimes called Negative Schizotypy, refers to a lack of close friends, constricted affect, excessive social anxiety, and also paranoid ideation. Finally, the Disorganized factor involves features such as odd or eccentric behaviour and speech. This conceptualization provides an important multidimensional opportunity for researchers since this factor structure resembles the symptoms reported in patients with schizophrenia^14^.

The literature proposes that the study of people presenting schizotypal personality traits may be useful to identify the core features of schizophrenia^15,16^. One of the main reasons is the reduction of the confounding conditions that may complicate the study of patients with schizophrenia, such as antipsychotic medication usage, social isolation, or recurrent hospitalization^17,18^. Moreover, regarding the field of individual differences, some authors think about schizotypy as being a non-clinical personality dimension aside from schizophrenia^19^, even highlighting the importance of studying schizotypy itself to develop an organising framework for social and affective sciences^20^.

Numerous studies exploring the influence of schizotypal personality traits have been centred on impairments in social, affective, and cognitive functions, suggesting that people presenting these personality traits may, in some way, reflect schizophrenia-like abnormalities, but milder in severity (for a review, see^20^). However, these research fields are extremely wide, and there are still many processes in which the possible influence of schizotypal personality traits has not yet been explored. In the present research, we aim to better understand the possible relation of these traits with one of these processes, which is the social learning of fear.

The experimental model of fear conditioning was derived from the seminal work of the physician Ivan Pavlov^21^, who was studying conditioning processes. When using this model, an initially neutral stimulus is contingently paired (*conditioned stimulus plus; *CS+) with an aversive stimulus (*unconditioned stimulus; US*), while a different stimulus is never paired (*conditioned stimulus minus; *CS−) with the US. Consequently, and if learning is achieved, this would result in a differential response to the different CSs. The CS+ alone is expected to generate a response (*conditioned response; CR*), similar to the one elicited by the US (*unconditioned response; UR*), while the CS− would be expected to result in a smaller response. That is, the former becomes a warning cue while the latter acts as a safety signal.

Some studies have explored this type of conditioning in patients with schizophrenia, apparently converging on the idea that, during a Pavlovian conditioning paradigm, these people show excessive physiological activity towards the CS−^22–24^, which reflects an anomaly in their acquisition of fear. Regarding schizotypy, a study using the Oxford-Liverpool Inventory of Feelings and Experiences (O-LIFE^25^) revealed that the Unusual Experiences factor was positively associated with a higher number of above-threshold Skin Conductance Responses (SCRs) when the CS− was presented and that both Unusual Experiences and Introvertive Anhedonia were negatively associated with the number of above-threshold SCRs when the CS+ was presented^26^. As far as we know, this is the only study exploring aversive Pavlovian conditioning in schizotypy. SCR is a phasic response to a stimulus in the electrodermal activity, and literature proposes it as a reliable measure to compare the activity of the autonomic nervous system towards different stimuli, being among the most widely employed indexes of conditioned fear responses^27,28^, with the US and CS+ usually generating a larger SCR than the CS−. Not being able to discriminate between stimuli as expected could be an indicator of abnormal conditioning processes, which could affect safety and risk learning as well as daily life.

Albeit direct experience is undoubtedly helpful to predict whether an aversive stimulus is likely to occur, humans and other social species can acquire conditioned fear through observational processes (for reviews, see^29,30^). This latter manner of learning entails a safer environment since the individual is not exposed to the US while learning the possible causes that may lead to it. Besides, this process shares important characteristics with direct fear learning, as well as similar neural mechanisms^29^, and has also been shown to be useful as an extinction procedure, yielding even better results than the standard extinction procedure, which has important implications from a clinical point of view^31^.

Even though more than half a century has passed since the first studies involving fear conditioning through observation^32,33^ and that researchers are still exploring aspects related to this issue (for reviews, see^34,35^), to our knowledge, it has not yet been explored if schizophrenia or schizotypal personality traits may affect this essential capacity. Thus, we believe that the study of the impact of schizotypal traits on the social learning of conditioned fear may suppose an important contribution to a better understanding of the underlying mechanisms that may regulate this process both in clinical and non-clinical populations. These traits have been genetically associated with the development of full-blown schizophrenia^36,37^, which would support their importance for the study of clinical populations, and it has been repeatedly demonstrated that schizotypal personality traits are also widely distributed across the general, non-clinical population (for a review, see^38^).

Therefore, the main goal of this research is to explore for the first time, as far as we know, whether the levels of schizotypal personality traits may have any influence in fear conditioning acquisition through social observation. Based on the results obtained in Balog et al.^26^, we expect to find abnormalities in SCR during observational fear conditioning in people with a high score in schizotypal personality traits. Concretely, these could be displayed both in a higher reactivity to the CS− due to a positive association of this measure with the Cognitive-perceptual factor, or in a lower reactivity to the CS+ due to a negative association of this measure with both the Cognitive-perceptual factor and the Interpersonal factor. It is worth noting that contrary to Balog et al.^26^, the participants will have no direct experience with the US at any moment during our procedure.

## Methods

### Participants

A total of 85 undergraduate students (40 men and 45 women) from the University of Almeria, ranging in age from 18 to 42 years (*M* = 22.2, *SD* = 3.8), with no history of psychiatric illness participated in the study. They all had a normal or corrected-to-normal vision and received one course credit for their collaboration. Data from 13 participants were excluded from the subsequent analyses because they firmly did not believe either that the model was receiving an electric shock during the learning phase or that they were going to receive an electric shock during the test phase (see Procedure for details). Therefore, the total sample used in the analyses was composed of 72 participants (34 men and 38 women) ranging in age from 18 to 34 years (*M* = 22.0, *SD* = 3.2). All participants gave written informed consent and were informed they could decide to stop the experiment at any moment. The study was approved by the Bioethics Committee in Human Research of the University of Almeria and was conducted following the Declaration of Helsinki.

### Instruments

#### Schizotypy assessment

The Schizotypal Personality Questionnaire (SPQ^39^) is a widely used tool for the assessment of schizotypal personality traits^11^. It comprises 74 items that refer to a broad range of features distributed across nine subscales, each containing seven to nine items answered dichotomously. Each “Yes” response yields a score of 1 and each “No” response yields a score of 0, so the total score in the questionnaire ranges from 0 to 74, with a higher score meaning a higher presence of schizotypal personality traits. Literature suggests that these nine subscales are better explained in a three-factor structure^12,40,41^. The Cognitive-Perceptual factor (α = 0.83) has a score ranging from 0 to 33 and integrates the four subscales Odd Beliefs or Magical Thinking, Unusual Perceptual Experiences, Ideas of Reference, and Suspiciousness. The Interpersonal factor (α = 0.88) has a score ranging from 0 to 33 and integrates the four subscales Social Anxiety, No Close Friends, Constricted Affect, and Suspiciousness. Finally, the Disorganized factor (α = 0.79) has a score ranging from 0 to 16 and integrates the two subscales Odd or Eccentric Behaviour and Odd Speech. The reliability for each factor was calculated on the set of items that compose each factor score. For this study, the Spanish validated version was used^42^.

#### Social fear learning task

**Figure 1:**
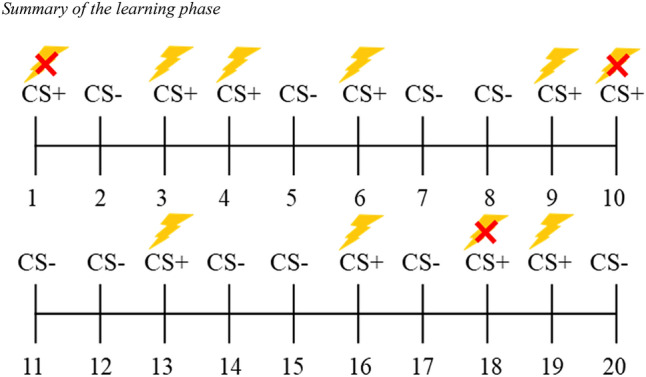
Summary of the learning phase. Stimuli sequence during the full presentation of the video. Trials of the CS+ that were paired with an electric shock are marked with a thunderbolt. Those CS+ that were not paired with an electric shock are marked with the same thunderbolt and a red cross. None of the CS− was paired with an electric shock.

Our procedure was adapted from Olsson et al.^43^. For the learning phase, we recorded a movie that showed a male model seated in front of a laptop with two electrodes attached to his right forearm. The screen of the laptop showed, in a pseudo-randomized order, 10 yellow squares and 10 blue squares which served as CSs. The stimuli size was 25.6 × 14.7 cm. Each colour could not appear more than two consecutive times. The coloured squares remained for 5 s on the screen, and between them, a blank screen with the word “Rest” was presented at the centre of the screen for 5 s. In the original video, 2.5 s after the onset of seven of the blue squares (see Fig. 1), the male model, who was a trained actor, simulated that he received an uncomfortable electric shock (US), twisting the hand that had the electrodes attached while making sounds complaining for approximately 1 s, after receiving a low-intensity shock for 0.5 s that served as a cue. The video was edited using Adobe After Effects^44^ to counterbalance which colour served as CS+ and CS− between subjects. Using the Biotrace + NX10 software^45^ we set markers at the onset of all the US and CS− for both versions of the video.

For the test phase, we designed another video that was similar to the one the male model was watching during the learning phase, but this time, participants directly saw the coloured squares on their screen. Again, 10 yellow squares and 10 blue squares were displayed in a pseudo-randomized order. The order of coloured squares was different from the employed on the learning phase but was the same for all participants. Please note that, as the colour of the CS+ has been counterbalanced among participants, the same colour could serve as CS+ for some participants and as CS− for others. Each colour could not appear more than two consecutive times. As in the learning phase, the coloured screens lasted for 5 s and, between them, a blank screen with the word “Rest” was shown for 5 s. Again, we set markers at the onset of all the CS+ and CS− using Biotrace + NX10^45^.

#### Skin conductance responses

SCRs were used to assess fear conditioning during both phases of the study. SCR is defined as a phasic autonomous response to a stimulus, usually measured as the increment in the Skin Conductance (SC) during a latency window (usually 0.5–5 s) following the stimulus onset^46^. This measure is the most commonly used to assess fear conditioning (for a full review of methods in fear conditioning, see^28^).

SC was measured through Ag–AgCl electrodes attached to the distal phalanges of the second and fourth digits of the non-dominant hand. The signal was amplified and recorded using the NeXus 10-MKII equipment and the Biotrace + NX10 software^45^. This software provides an index of the raw SC at a rate of 32 samples per second. These indexes were then processed using continuous decomposition analysis (CDA). CDA divides the raw SC into continuous signals of both the slow-varying tonic activity, or Skin Conductance Level (SCL), and the fast-varying phasic activity, or SCR, which is the measure we analyse in the present research. As an index for the SCR, we considered the time integral (integrated SCR; ISCR) of the deconvoluted phasic activity for each stimulus within a response window of 0.5–5 s post-stimulus. Following Benedek and Kaernbach^47^, this scoring method decreases biases due to superimposing SCRs and also considers the continuous shape of phasic responses, which may be preferable to the simple consideration of local minima and maxima. Data was also pre-processed applying a Butterworth low-pass filter (5 Hz) and entered into a CDA with two optimization runs for each participant. A minimal amplitude threshold of 0.01 μS was also set. All this was achieved employing the software Ledalab^47^.

### Procedure

Participants were tested individually in a quiet room that was the same shown in the video of the learning phase, using an Asus ROG GL552VW laptop with a 15.6″ screen. In the beginning, they were told they were about to see a video of a person performing an experiment similar to the one they were going to do afterwards. They were also told they could stop the experiment at any time. A trained researcher, who was present in the room during the whole experiment, attached the equipment used to obtain the physiological data to the non-dominant hand of the participant. After verifying that the equipment was recording correctly, the video that was designed for the learning phase was started. Aside from the previously mentioned equipment, a Blood Volume Pulse sensor was attached to the distal phalanx of the middle finger of our participants, for purposes out of the scope of this study. Although participants had been previously informed about the experiment, the following text (in Spanish) was shown in the first 30 s of the video:

You are going to see a video of a person doing an experiment similar to the one you are about to perform. The person of the video receives electric shocks paired with one of the two squared colours that are presented. Pay attention to the video because in the experiment you are going to do afterwards, you are going to receive electric shocks paired with the same coloured square as the person of the video.

This video lasted 4 min and 25 s and, once it ended, the trained researcher attached two new electrodes that could deliver electric shocks to the forearm of the dominant arm of the participant. These electrodes were connected to a stimulus isolator and were the same the participants had watched in the previous video. Participants were reminded that, in this second phase of the task, they were about to receive electric shocks paired with the same coloured square as the person they had just seen. They were told that they were only going to receive between one and three electric shocks and that their intensity would be lower. Importantly, no shocks were administered to the participants to ensure that fear learning was attained only through social means. Once the participant was ready, the researcher started the second video which began by showing the next instructions (in Spanish) on the screen for 20 s:

Now it is your turn. You are going to see coloured squares similar to those included in the video you have just seen but in a different order. You are going to receive electric shocks paired with the same colour you observed on the previous video, with the difference that the person of the previous video received seven electric shocks and you are only going to receive between one and three. There will be no electric shocks while the other coloured square is shown or during the breaks.

This last video lasted 3 min and 54 s. This second video was shorter because it just showed the different coloured screens and not the researcher attaching the equipment to the male model that simulated to receive electric shocks before the experiment started. However, the duration of the experiment itself (the sequence of screens) was the same. Once this video ended, the researcher informed the participant that he/she had to complete the SPQ^39^ and that it was mandatory to answer all the items. We decided to assess participants at the end of the task because the SPQ^39^ includes items that involve aspects such as social anxiety or suspiciousness, which could prime the participants into these feelings. Finally, the researcher asked the participant whether he/she had believed that electric shocks were being administered to the male model of the first video and if he/she have thought that any electric shocks were going to be administered to him/her. Data from participants who answered “No” to any of these questions (n = 13) was removed from subsequent analyses.

## Results

### Schizotypy assessment: descriptive statistics

In order to describe the distribution of schizotypal personality traits in our sample, we calculated the mean, the standard deviations, the range, the skewness, the kurtosis, the median, and some other percentiles (10, 25, 75, and 90) separately for each of the three factors and the total score. These descriptive statistics of the SPQ^39^ scores are reported in Table 1.

**Table 1:** Means, standard deviations, ranges, skewness, kurtosis, and percentiles of the scores of each factor and the total score of the SPQ obtained by participants.

Factor	Mean	SD	Range	SK	K	Percentiles				
						10	25	50	75	90
Cognitive-Perceptual	9.3	5.5	0–23	0.40	− 0.75	3.0	5.0	8.0	14.0	16.0
Interpersonal	13.0	6.8	1–32	0.33	− 0.19	5.0	7.8	13.1	17.0	21.0
Disorganized	5.0	3.5	0–13	0.39	− 0.82	1.0	2.8	5.3	8.0	10.0
Total score	24.7	10.5	7–51	0.23	− 0.70	11.0	15.0	26.0	31.3	37.9

SD, standard deviation; SK, skewness; K, kurtosis.

### Analyses of the SCRs

SCRs for each stimulus were estimated as the time integral (integrated SCR; ISCR) of the deconvoluted phasic activity for each stimulus within a response window of 0.5–5 s post-stimulus. As this index is provided in μS and thus it may present a high interindividual variability^27^, we followed the recommendations in Ben-Shakhar^48^ to correct these differences. For each participant, the ISCRs generated by each stimulus in each of the phases were standardized separately. After that, we averaged responses across several trials to try to compensate for the usual positively skewed distributions observed in single trials, which is mainly generated due to non-responses^28^. For those interested, trial-by-trial plots and a report of the distributions of all the dependent variables are provided in Supplementary Material.

In order to perform these statistical analyses, we decided to use the robust generalization of the Welch–James statistic^49–52^ proposed in Keselman et al.^53^ for the analysis of variance and posthoc comparisons, which does not pool across heterogeneous sources of variability and estimates the error degrees of freedom from the sample data. Additionally, we also used robust measures of central tendency and variability, such as trimmed means and winsorized variances, since they generally overcome the biasing effects of non-normality more convincingly than classical estimators^54,55^. To estimate these values using the widest range of our data, the amount of symmetric trimming was set to 10% since some authors have reported it to be a suitable choice for dealing with non-normal data^56^. An empirical critical value was also estimated for the hypothesis testing using bootstrapping^57^, so *p*-values shown in the results section are adjusted accordingly. To simplify the reading of the article, only significant results will be commented on.

All the following analyses were performed using the “welchADF” package^58^, and the “WRS” package^55^, both available for the free “R” software^59^. All the plots were also designed with the free “R” software^59^, using the “ggplot2” package.

#### Learning phase

In the learning phase, for the US, we averaged the responses generated by all the seven USs. Regarding the CS−, all but the first CS− were averaged, because the first time the CS− is presented it is still not a safety cue since the first CS+ -US association occurs in the third trial (which corresponds to the second CS+). Finally, CS+ data were not analysed because most of the CS+ were followed, after 2.5 s, by the US. As the latency window used for analysing the physiological measurements ends 5 s after the onset of the stimulus, using these measurements could lead to superimposition, distorting the measurement^27^. In this learning phase, we were more interested in replicating the difference in the SCRs generated by the US and the CS− previously reported in the literature^43^. Stimulus (US and CS−) was taken as a within-subject variable and sex (men and women) as a between-subject variable. Although we did not have any hypotheses concerning sex, we wanted to be sure that it did not have any influence on the task, since the observed person in the learning phase was a male.

**Figure 2:**
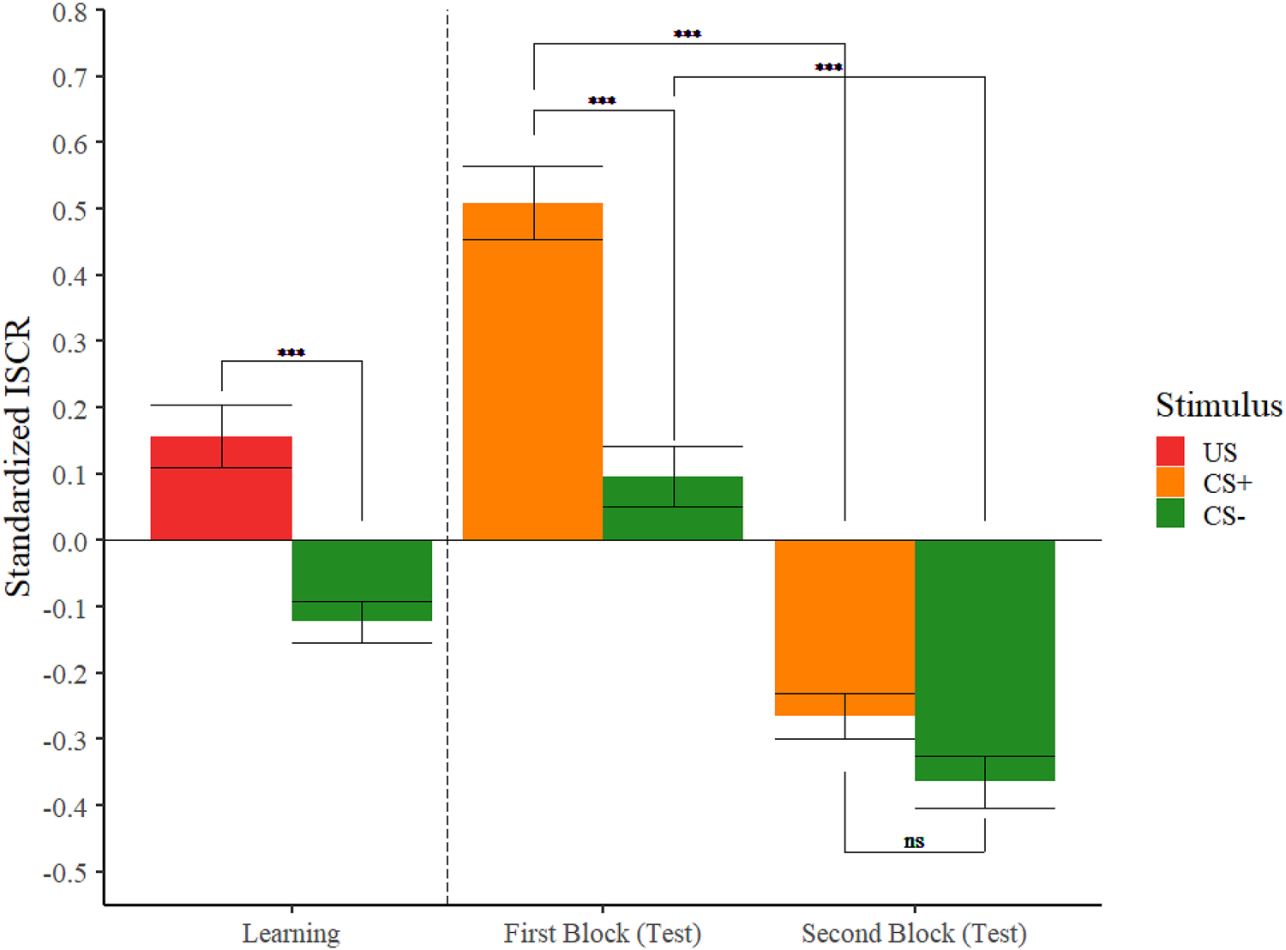
Standardized ISCRs as a function of stimulus and phase. Trimmed means (10%) of the standardized ISCRs of our participants as a function of stimulus in the learning phase and both blocks of the test phase. Error bars represent the standard error of the trimmed mean. The three asterisks (***) indicate a significant difference *p* < 0.001. The letters (ns) indicate a non-significant difference *p* > 0.05.

The omnibus contrast using the Welch-James statistic revealed that the only significant effect was the main effect of stimulus, *T*_WJ_(1, 56.2) = 15.07, *p* < 0.001, that is, the US generated larger SCRs than the CS−, $\widehat{\updelta }$_R_ = 1.66 (95% CI [0.83, 2.61]). These results can be observed in Fig. 2.

#### Test phase

For the test phase, we divided the task into two blocks, including the first five CS+ and the first five CS− in the first block, and the last five CS+ and the last five CS− in the second block. This way, we could discriminate the physiological responses during the initial and the later stages of the test phase. This is especially relevant since previous studies have found differences in the SCR between patients with schizophrenia and control participants using a fear conditioning paradigm just in the latter stages of the conditioning phase^24^. Stimulus (CS+ and CS−) and block (first and second) were taken as within-subject variables and sex (men and women) as a between-subject variable.

The omnibus contrast using the Welch-James statistic revealed significant main effects of stimulus, *T*_WJ_(1, 57.4) = 23.02, *p* < 0.001, and block, *T*_WJ_(1, 56.3) = 157.00, *p* < 0.001, which were modulated by the significant Stimulus × Block interaction *T*_WJ_(1, 57.9) = 11.00, *p* = 0.002. This interaction revealed that the CS+ generated larger SCRs than the CS− in the first block, *t*_WJ_(58.0) = 23.51, *p* < 0.001, $\widehat{\updelta }$_R_ = 1.96 (95% CI [1.15, 2.86]), but not in the second block, *t*_WJ_(57.3) = 3.37, *p* = 0.072, $\widehat{\updelta }$_R_ = 0.637 (95% CI [− 0.03, 1.38]). On the other hand, the SCRs generated by the CS+ were higher in the first block than in the second block, *t*_WJ_(57.8) = 116.4, *p* < 0.001, $\widehat{\updelta }$_R_ = 4.15 (95% CI [3.19, 5.12]). Similarly, the SCRs generated by the CS− were also higher in the first block than in the second block, *t*_WJ_(56.3) = 50.37, *p* < 0.001, $\widehat{\updelta }$_R_ = 2.55 (95% CI [1.69, 3.51]). These results can be observed in Fig. 2.

#### Influence of schizotypal personality traits on SCRs

To check whether any of the three factors of schizotypal personality, as well as the total score, had any direct influence on the SCRs applying a linear regression model, we used the approach recommended by Wilcox^55^ based on the 0.632 estimator. It uses bootstrapping to estimate the prediction error of every predictor variable, in isolation and in combination with the others, as well as the prediction error of a null model that would not use any predictor. This analysis was performed with both the ordinary least squares estimator and the Theil–Sen estimator^60,61^ since this latter has been reported to substantially increase the probability of identifying the best model under circumstances in which parametric assumptions are not met^62^. We repeated the analysis separately for every dependent variable, which was the averaged physiological response for every stimulus in the learning phase (US and CS−), and for every stimulus in the first and second blocks of the test phase (CS+ and CS−), choosing the three factors and the total score of the SPQ^39^ as predictor variables.

The 0.632 estimator revealed that the null models always showed a lower prediction error than models that included any of the schizotypal personality factors, both in isolation and in combination, for all the dependent variables, using both the ordinary least squares estimator and the Theil–Sen estimator. The total score in the SPQ was not a better predictor than the null model either. Thus, none of these variables was a reliable predictor of the SCRs. These results are shown in Table 2.

**Table 2:** Prediction errors based on the 0.632 estimator. Different possible models when trying to predict the standardized ISCRs generated by each stimulus in the learning phase and both blocks of the test phase using the ordinary least squares estimator (and the Theil-Sen estimator). The model with the smallest prediction error is written in boldface. CP, Cognitive-Perceptual Factor; IN, Interpersonal Factor; DI, Disorganized Factor.

Predictors	Learning		First block (Test)		Second block (Test)	
	US	CS−	CS+	CS−	CS+	CS−
CP	0.3348 (0.3369)	0.2257 (0.2303)	0.3721 (0.3818)	0.3118 (0.3144)	0.2481 (0.2482)	0.2642 (0.2633)
IN	0.3386 (0.3480)	0.2266 (0.2322)	0.3721 (0.3795)	0.3162 (0.3172)	0.2485 (0.2483)	0.2622 (0.2607)
DI	0.3387 (0.3451)	0.2283 (0.2317)	0.3690 (0.3774)	0.3049 (0.3047)	0.2477 (0.2498)	0.2633 (0.2626)
CP + IN	0.3442 (0.3473)	0.2311 (0.2367)	0.3797 (0.3871)	0.3192 (0.3189)	0.2561 (0.2552)	0.2696 (0.2668)
CP + DI	0.3446 (0.3460)	0.2329 (0.2365)	0.3782 (0.3842)	0.3100 (0.3061)	0.2540 (0.2546)	0.2677 (0.2666)
IN + DI	0.3485 (0.3520)	0.2350 (0.2383)	0.3774 (0.3815)	0.3152 (0.3121)	0.2545 (0.2555)	0.2684 (0.2668)
CP + IN + DI	0.3538 (0.3525)	0.2397 (0.2425)	0.3858 (0.3872)	0.3198 (0.3154)	0.2622 (0.2600)	0.2759 (0.2719)
Total SPQ score	0.3367 (0.3421)	0.2272 (0.2314)	0.3735 (0.3827)	0.3108 (0.3100)	0.2476 (0.2475)	0.2598 (0.2605)
Null model (no predictors)	**0.3289** **(0.3289)**	**0.2201** **(0.2201)**	**0.3589** **(0.3589)**	**0.3044** **(0.3044)**	**0.2397** **(0.2397)**	**0.2593** **(0.2593)**

Furthermore, we considered important to check whether the possible differences found in SCRs between each pair of observations (e.g., the CS+ and the CS− in the first block) could be explained by the presence of schizotypal personality traits. In order to do these tests, we employed the method DY^55^, a generalization of Yuen’s test^63^ that compares the possible effect that a covariate may exert towards the differences between pairs of observations of the same participant. Besides, this method can deal with curvature as it does not make any assumption about the shape of the regression lines, and it provides a confidence interval for the differences, as well as a critical *p*-value to control the probability of Type I errors in multiple pairwise comparisons. This is done by choosing *i* covariate values *x*_*i*_, and comparing the scores in the target pair of observations of participants with a score close to *x*_*i*_ in the chosen covariate while using this method. With the aim of getting the widest range of scores of each factor of schizotypy while keeping the samples as large as possible, we manually set these values to 3 (n = 37), 7 (n = 46), 10 (n = 46), 13 (n = 36), and 17 (n = 25) for the Cognitive-Perceptual factor; to 5 (n = 34), 9 (n = 50), 13 (n = 51), 17 (n = 49), and 21 (n = 33) for the Interpersonal factor; to 2 (n = 48), 4 (n = 56), 6 (n = 54), 8 (n = 44), and 10 (n = 34) for the Disorganized factor; and to 17 (n = 50), 22 (n = 49), 27 (n = 46), 32 (n = 42), and 37 (n = 37) for the total score. The same participant could be included in more than one group. For example, a participant with a score of 5 in the Cognitive-perceptual factor would be included in the comparisons for *x*_*i*_ values 3 and 7, but not for the *x*_*i*_ values 13 and 17. This analysis was repeated for every possible pair of stimuli in each of the phases, using each factor of schizotypy as a covariate, one at a time, and setting a symmetric trimming of 10% to account for the possible biasing effects of non-normality^56^. To simplify the reading of the paper, only those tests that clearly suggested variations in the differences between pairs of stimuli depending on schizotypal personality traits will be commented on.

**Figure 3:**
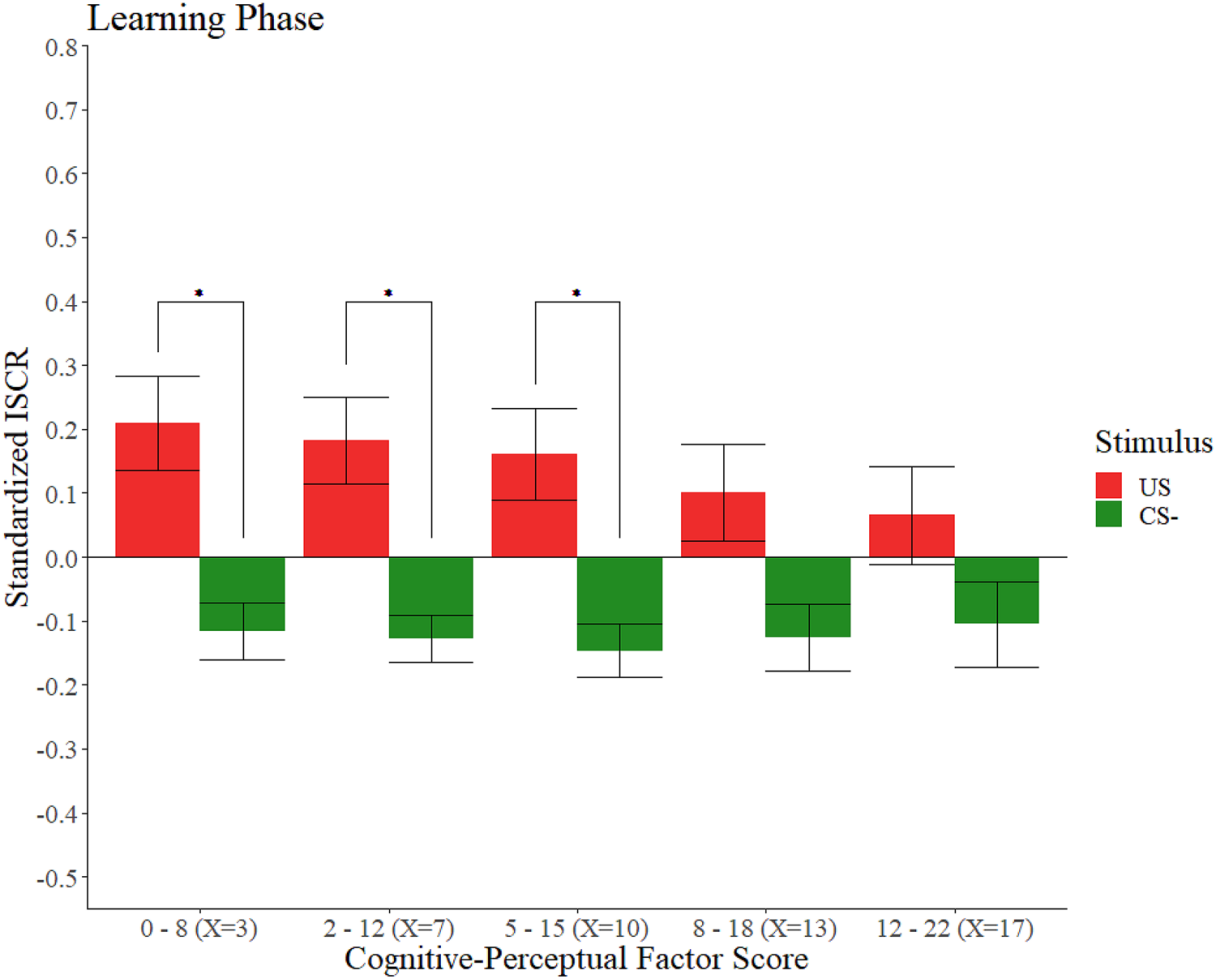
Standardized ISCRs as a function of stimulus and the Cognitive-Perceptual factor score in the learning phase. Trimmed means (10%) of the standardized ISCRs of our participants as a function of stimulus and their score in the Cognitive-Perceptual factor in the learning phase. Error bars represent the standard error of the trimmed mean. The asterisks (*) indicate adjusted significant differences (*p* < 0.01).

The use of the method DY revealed that the differences between pairs of observations of the participants varied depending on their schizotypal personality traits. During the learning phase, participants presenting low scores in the Cognitive-Perceptual factor exhibited larger SCRs when watching the US than when watching the CS−. Nevertheless, those participants with higher scores in the Cognitive-Perceptual factor did not show any significant differences in their SCRs when watching the US and the CS−. These results are presented graphically in Fig. 3. Neither the rest of the factors nor the total SPQ score did show any clear pattern and, contrary to what is shown in those participants with high scores in the Cognitive-Perceptual factor, their 95% confidence intervals for the differences in SCRs between US and CS− never contained the 0 value within its limits, always suggesting a higher SCR when watching the US than when watching the CS−. All the corresponding statistics are shown in Table 3.

**Table 3:** Pairwise comparisons between the standardized ISCRs generated by stimuli in the learning phase as a function of the score (X) in the three factors of the SPQ as well as the total SPQ score.

Factor	X (n)	US vs. CS− (Learning phase)			
		Dif [95% CI]	SE	*Ty*	*p*
Cognitive-Perceptual Factor	3 (37)	**0.325 [0.114, 0.536]**	**0.103**	**3.15**	**0.004**
	7 (46)	**0.311 [0.122, 0.499]**	**0.093**	**3.34**	**0.002**
	10 (46)	**0.307 [0.099, 0.515]**	**0.103**	**2.99**	**0.005**
	13 (36)	0.227 [− 0.010, 0.464]	0.116	1.96	0.060
	17 (25)	0.171 [− 0.094, 0.436]	0.127	1.34	0.194
Interpersonal Factor	5 (34)	0.270 [0.044, 0.496]	0.110	2.15	0.021^a^
	9 (50)	0.218 [0.027, 0.408]	0.094	2.32	0.026^a^
	13 (51)	**0.312 [0.129, 0.496]**	**0.091**	**3.44**	**0.001**
	17 (49)	0.233 [0.038, 0.429]	0.097	2.41	0.021^a^
	21 (33)	**0.374 [0.134, 0.614]**	**0.117**	**3.20**	**0.004**
Disorganized Factor	2 (48)	**0.319 [0.125, 0.513]**	**0.096**	**3.32**	**0.002**
	4 (56)	**0.281 [0.109, 0.453]**	**0.085**	**3.29**	**0.002**
	6 (54)	0.217 [0.053, 0.381]	0.081	2.67	0.011^a^
	8 (44)	**0.302 [0.119, 0.486]**	**0.090**	**3.35**	**0.002**
	10 (34)	**0.264 [0.077, 0.452]**	**0.091**	**2.89**	**0.007**
Total SPQ score	17 (50)	**0.274 [0.080, 0.468]**	**0.096**	**2.86**	**0.007**
	22 (49)	0.245 [0.037, 0.454]	0.103	2.38	0.022^a^
	27 (46)	**0.325 [0.107, 0.544]**	**0.108**	**3.01**	**0.005**
	32 (42)	0.279 [0.052, 0.506]	0.111	2.50	0.017^a^
	37 (37)	**0.308 [0.089, 0.526]**	**0.107**	**2.87**	**0.007**

Adjusted significant differences (*p* < 0.01) are boldfaced. SE, Standard Error of the difference.

^a^95% CI does not include 0 and adjusted significance level (*p* < 0.01) is not reached.

**Figure 4:**
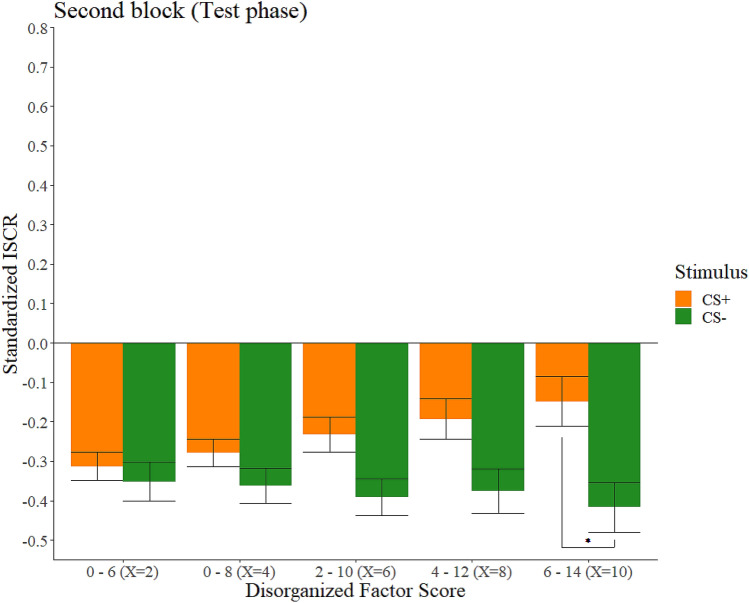
Standardized ISCRs as a function of stimulus and Disorganized factor score in the second block of the test phase. Trimmed means (10%) of the standardized ISCRs of our participants as a function of stimulus and their score in the Disorganized factor in the second block of the test phase. Error bars represent the standard error of the trimmed mean. The asterisk (*) indicates adjusted significant differences (*p* < 0.01).

In the first block of the test phase, participants with the lowest scores in the Cognitive-Perceptual factor and participants with the highest scores in the Interpersonal factor, were the only ones whose differences between their SCRs when watching the CS+ and when watching the CS− did not reach the adjusted significance level (*p* = 0.016 and *p* = 0.021, respectively). Nevertheless, 95% confidence intervals for these differences did not contain the 0 value within its limits, suggesting a higher SCR for the CS+ than for the CS−. In the second block of the same phase, only the group of participants with the highest scores in the Disorganized factor reached the adjusted significance level for the differences in SCRs when watching the CS+ and when watching the CS− (*p* = 0.006). These results are represented graphically in Fig. 4. The Cognitive-perceptual factor and the Interpersonal factor suggest a similar trend, since groups with the highest scores show that the 95% confidence intervals for their differences between stimuli did not include the 0 value, but the significance level (0.01) is not reached at any of the points for these factors. Statistics involving these effects are shown in Table 4.

**Table 4:** Pairwise comparisons between the standardized ISCRs generated by stimuli in the first and the second block of the test phase as a function of the score in the three factors of the SPQ (X). Adjusted significant differences (p < .01) are boldfaced. SE, Standard Error of the difference. a, 95% CI does not include 0 and adjusted significance level (p < .01) is not reached.

Factor	X (n)	CS+ vs. CS− (1st block of the test phase)				CS+ vs. CS− (2nd block of the test phase)			
		Dif [95% CI]	SE	*Ty*	*p*	Dif [95% CI]	SE	*Ty*	*p*
Cognitive-Perceptual Factor	3 (37)	0.305 [0.060, 0.551]	0.120	2.54	0.016^a^	0.036 [− 0.132, 0.203]	0.082	0.435	0.667
	7 (46)	**0.387 [0.165, 0.609]**	**0.110**	**3.53**	**0.001**	0.024 [− 0.132, 0.181]	0.077	0.314	0.755
	10 (46)	**0.384 [0.171, 0.597]**	**0.105**	**3.65**	**0.000**	0.092 [− 0.046, 0.229]	0.068	1.35	0.185
	13 (36)	**0.525 [0.293, 0.757]**	**0.113**	**4.63**	**0.000**	0.117 [− 0.034, 0.267]	0.074	1.58	0.124
	17 (25)	**0.511 [0.201, 0.821]**	**0.148**	**3.44**	**0.003**	0.173 [0.036, 0.311]	0.066	2.63	0.016^a^
Interpersonal Factor	5 (34)	**0.434 [0.169, 0.700]**	**0.129**	**3.36**	**0.002**	− 0.010 [− 0.181, 0.161]	0.083	− 0.122	0.903
	9 (50)	**0.400 [0.188, 0.612]**	**0.105**	**3.81**	**0.000**	0.017 [− 0.102, 0.136]	0.059	0.287	0.776
	13 (51)	**0.371 [0.169, 0.573]**	**0.100**	**3.71**	**0.001**	0.051 [− 0.063, 0.167]	0.057	0.906	0.370
	17 (49)	**0.320 [0.122, 0.518]**	**0.098**	**3.26**	**0.002**	0.117 [− 0.001, 0.234]	0.058	2.01	0.051
	21 (33)	0.294 [0.049, 0.539]	0.119	2.47	0.021^a^	0.291 [0.027, 0.318]	0.071	2.44	0.022^a^
Disorganized Factor	2 (48)	**0.338 [0.136, 0.540]**	**0.100**	**3.38**	**0.002**	0.039 [− 0.086, 0.164]	0.062	0.63	0.532
	4 (56)	**0.408 [0.216, 0.600]**	**0.096**	**4.27**	**0.000**	0.083 [− 0.037, 0.203]	0.060	1.39	0.172
	6 (54)	**0.471 [0.271, 0.670]**	**0.099**	**4.76**	**0.000**	0.158 [0.022, 0.294]	0.067	2.35	0.023^a^
	8 (44)	**0.437 [0.194, 0.681]**	**0.120**	**3.65**	**0.000**	0.183 [0.018, 0.348]	0.081	2.25	0.031^a^
	10 (34)	**0.512 [0.212, 0.812]**	**0.146**	**3.50**	**0.002**	**0.268 [0.082, 0.455]**	**0.091**	**2.95**	**0.006**

Lastly, in the test phase, regarding the CS+ and the CS−, the method DY suggested that all the groups of participants presented a higher SCR when watching these stimuli in the first block than when watching them in the second block, regardless of their score in the different factors of the SPQ. These results are reported in the Supplementary Material.

Additionally, as some studies have suggested that men and women score differently in schizotypal personality traits^64^, we wanted to check whether the proportion of men and women was similar in each group. We performed a chi-squared test in each of the groups. All the groups had a similar proportion of men and women (all *p*s > 0.05) except for the one comprised of those with the lowest scores in the Disorganized factor (*x*_*i*_ = 2), which included more women than men (n_men_ = 17, n_women_ = 31, *χ*^2^ = 4.08, *p* = 0.043). All these analyses are provided in Supplementary Material. For those interested, a full report of these analyses and the rest of the plots and statistics using the method DY are also available in Supplementary Material.

## Discussion

The main goal of the present study was to explore for the first time, as far as we know, the role that schizotypal personality traits may play in fear conditioning acquisition through social observation. As it has been previously observed in similar research^43^, our participants exhibited a larger SCR when watching the US than when watching the CS− during the learning phase, and a larger SCR when they were presented with the CS+ than when they were presented with the CS− in the test phase. This latter effect was only noticeable in the first block of the task but not in the second block. This reflects the acquisition of a conditioned fear response during the learning phase at the beginning of the test phase and also suggest the extinction of this response after several non-reinforced trials, which is in accordance with the classical conditioning theory (e.g., Rescorla and Wagner^65^). It is interesting that, even though our participants were told they were going to receive between one and three electric shocks, they stopped showing a higher SCR when they saw the CS+ than when they saw the CS− during the second block of the test phase. This effect may not be explained by our participants believing they were not going to receive any electric shocks, because those participants were excluded from the analyses.

Based on previous results studying direct aversive conditioning in people with schizotypal personality traits^26^, we expected to find a higher reactivity to the CS− in the participants with higher scores in the Cognitive-perceptual factor and lower reactivity to the CS+ in those with lower scores in the Cognitive-perceptual factor and/or the Disorganized factor. None of these effects have been directly replicated in our study. The models using the scores of the different factors of the SPQ^39^ as predictors, both in combination and in isolation, as well as those using the total score of the questionnaire, presented a higher prediction error than the null models that used none of them. Thus, as none of these variables was a reliable predictor of the SCRs, we think it would not be methodologically adequate to build a model upon predictors that may yield a higher prediction error than a null model. However, the exploration of the pairwise comparisons of the SCRs to different stimuli depending on schizotypal personality traits revealed some interesting results.

On the one hand, in the learning phase, our results highlighted a relevant effect regarding the Cognitive-Perceptual factor. Specifically, participants with low scores in this factor presented, as expected, significant differences between their SCRs toward the US and the CS−, with the US generating a significantly larger SCR. However, participants with higher scores did not show this significant difference, that is, they were equally responsive to both stimuli.

Previous researches have reported that patients with schizophrenia do not show differential SCRs to an aversive social stimulus during the acquisition phase, suggesting an intrinsic impairment of the Pavlovian conditioning in associative learning in schizophrenia^23^. Even though they measure the differential SCR to the CS+ and the CS−, we consider it highly interesting to have obtained similar results in our social fear conditioning experiment depending on the level of Cognitive-Perceptual schizotypal personality traits. Remarkably, Balog et al.^26^ study already found anomalies in direct aversive conditioning depending on the score in the Unusual Experiences factor. Even though our approach is different, we consider this result to be relevant in order to better understand the relationship between positive schizotypy and fear learning processes.

Crucially, even though during the learning phase these participants with high scores did not show a significant difference in their SCRs when they watched the US and the CS−, they showed the expected conditioned response in the first block of the test phase. Some studies have proposed social fear learning as a model of empathy^66^, so another possibility is that this lack of differences between the SCRs generated by both stimuli is due to a lack of empathy. It is possible that watching a person receiving an electric shock is not sufficiently affecting these people, but when they are told they are going to receive electric shocks paired with the same colour, they are sufficiently affected to respond differently to the CS+ and the CS−. A study of social fear learning^67^ related emotional empathy with a higher SCR when watching the US during the observational phase in the group in which the instructions made explicit that the person in the video was going to receive an electric shock. Additionally, a recent study reported that positive schizotypal traits were significantly and negatively related to empathy for other’s pain using Evoked Response Potential (ERP) measurements^68^, which is in line with this finding, suggesting a lack of responsivity when watching a person receiving an electric shock. Future studies are needed to further explore the modulation of the SCR during the observational phase of social fear learning experiments by the Cognitive-perceptual factor, and whether the impairments of the Pavlovian conditioning processes are affected in a similar way to what happens in people with schizophrenia with the differential SCR to the CS+ and the CS−^23^.

Regarding the possible effect of the rest of the schizotypal personality factors in the learning phase of our task, we consider it is important to highlight the relative complexity of our analyses involving the method DY. As this method aims to detect the variations in the differences found between pairs of observations along a continuum, results at the different points of the continuum cannot be taken into account isolatedly, and a holistic view about what happens at all the different points of the continuum is needed to make a conclusion. Furthermore, in line with some authors^69–71^, making a dichotomous decision based only on a *p*-value may lead us to lose important information, so they advise to additionally use different estimators, such as confidence intervals and effect sizes. For this reason, we consider that, as none of these groups show a clear pattern, since the 95% confidence intervals for their differences between stimuli never include the 0 value and always suggest a higher SCR when watching the US than when watching the CS−, which is the expected response, it would not be advisable to discuss these results based only on the adjusted significance level, which is reached in some groups, but not in all of them (all *p*s < 0.026). Thus, we may not make any inferences about the effect of the rest of the schizotypal personality factors in the learning phase of our task.

On the other hand, during the test phase, all groups of participants presented adjusted significant differences between their SCRs towards the CS+ and the CS− in the first block, except for those with the lowest scores in the Cognitive-Perceptual factor and those with the highest scores in the Interpersonal factor. Similar to what was mentioned above, when looking to the 95% confidence intervals for their differences, we see that the 0 value is never included on them and that the values within them suggest a higher SCR for the CS+ than for the CS−. For this reason, we also consider that the evidence about the possible effect of these factors in this first block is not strong enough to draw any conclusions.

Nevertheless, in the second block, only the group of participants with the highest scores in the Disorganized factor maintained a significantly higher SCRs when watching the CS+ than when watching the CS−, suggesting that they were the only ones that kept showing a differential response as a function of the stimuli in the latter stages of the task. To explain these results, we think it is needed to focus on the extinction process. Rescorla and Wagner^65^ suggested that extinction learning occurs when there is a mismatch between a high US expectancy and a low actual rate in which the US occur, and called this process prediction error, which is responsible for a correct updating of these learning processes. In our test phase, the US does never occur, so participants should show a similar SCR towards both CSs in the latter stages of the tasks if their extinction mechanisms are not impaired, since the US expectancy should decay along the test phase. An aberrant prediction error, generated from associative learning deficits similar to those presented in schizophrenia (see Corlett et al.^72^), might involve an underlying impairment in fear extinction in those participants scoring high in the Disorganized factor. For instance, it might be that they had anomalous expectancy ratings for the US or that they do not update this expectancy in the same way as people with less Disorganized schizotypal traits do. Regarding the first, Dibbets et al.^73^ found that people scoring high in anxiety traits rated the US expectancy to the CS+ as higher than participants with low anxiety traits in the latter stages of their extinction phase. Interestingly, the Disorganized factor of schizotypy has been related to anxiety traits with a medium effect size^74^. This suggests that it could be relevant to replicate our study measuring trial-by-trial US expectancy to clarify the mechanisms underlying the effect of this factor on the fear of extinction processes, since our task was not originally designed as an extinction paradigm. Further studies could also explore whether something similar occurs in other non-social conditioning procedures including an extinction phase with an analytical approach similar to ours. The fact that extinction processes could be impaired in people with high levels of these traits may be interesting to explain their relationship with anxiety^74^.

The Cognitive-Perceptual and the Interpersonal factors suggest a similar trend, since even though the groups with the highest scores in both factors fail to reach the adjusted significance level, their 95% confidence intervals for the differences between stimuli did not include the 0 value and suggest a higher response towards the CS+ than towards the CS− also on the second block. Although we cannot make any inferences based on these differences since they are non-significant, it should be noted that significant positive correlations of both factors with anxiety have also been found^75^. As this is the first study looking into these issues in a social fear learning task in people as a function of their levels of schizotypal personality traits, we consider that a more extensive inspection of the extinction processes in future studies is needed to make a clear conclusion about the effect of these factors.

In conclusion, our study explores, for the first time, the effect of schizotypal personality traits in social fear learning processes. This effect was observed during both the learning and the test phases. In the learning phase, we have seen that participants with low scores on the Cognitive-Perceptual factor had a significantly higher reactivity when the US was presented compared to the CS−, while participants with higher scores on this factor did not show this effect. On the other hand, in the test phase, the effect of schizotypal personality traits was expressed again concerning the Disorganized factor. Only those participants with the highest scores on this factor maintained a significantly higher SCR when watching the CS+ than when watching the CS− in the second block of the test phase, probably suggesting an impairment in their extinction processes.

However, these results have to be taken cautiously since our study presents some limitations. Firstly, no a priori hypotheses were made that could predict our results at the moment it was carried out. Future research is needed to replicate and confirm these findings, as well as the possible effect of schizotypal personality traits on expectancy ratings for the US. Secondly, more information about the sample could have been collected. Participants could have been assessed about aspects such as substance use and abuse or family history of psychiatric illness, as well as about other potential covariates such as Intelligence Quotient or socio-economic status. It would have been desirable to have these variables registered to ensure the replicability of the study. Thirdly, our study does not include people from clinical populations to make comparisons. Studies using clinical populations such as people diagnosed with schizophrenia or with schizotypal personality disorders are encouraged. In those studies, we expect larger and more remarkable effects to be present, which would be endorsed by and in line with our results. Finally, the timing of the US and the rest periods of our experiment could have affected our measurements. The fact that in the learning phase the US was administered 2.5 s after the presentation of the CS+ may suppose that we could not be measuring the response to the CS+ in isolation during the test phase, but also a response to an apparently absent US. Regarding the rest periods, it is recommended to leave at least 6 s to allow a return to baseline of the SCL^28^, while ours are just 5 s. Even though these are systematic errors that should equally affect all our participants, and that the methods we employ (CDA) decrease the bias generated by superimposition^47^, future studies may replicate this finding employing standardized methodologies for evaluating social fear acquisition^76^.

## Supplementary Information

Supplementary Information.

## Data Availability

The datasets generated during and/or analysed during the current study are available from the corresponding author on reasonable request.
