# Supplementary material for: Schizotypal personality traits and the social learning of fear
Source: Sci Rep. 2021 Nov 29;11:23048. doi: 10.1038/s41598-021-02336-6 (PMC8630166; doi:10.1038/s41598-021-02336-6)
Supplement: Supplementary file 1 — Supplementary Information. [file 41598_2021_2336_MOESM1_ESM.docx]

| **Supplementary Table 1**  Means, standard deviations, ranges, skewness, kurtosis, percentiles, and tests of normality and homoscedasticity of the standardized Integrated SCRs (ISCRs) of the participants in each phase. | | | | | | | | | | | | | | | | | |
| --- | --- | --- | --- | --- | --- | --- | --- | --- | --- | --- | --- | --- | --- | --- | --- | --- | --- |
|  |  |  |  |  |  |  | Percentiles | | | | |  | Normality | |  | Homoscedasticity | |
| Learning phase |  | Mean | SD | Range | SK | K | 10 | 25 | 50 | 75 | 90 |  | *W* | *p* |  | *F* | *p* |
| US | Men | .21 | .38 | -.63 - .1.18 | .13 | .24 | -.22 | -.01 | .20 | .45 | .59 |  | .98 | .91 |  | 1.09 | .30 |
|  | Women | .14 | .43 | -.48 - .1.04 | .54 | -.77 | -.33 | -.18 | .03 | .40 | .74 |  | .94 | .05* |  |  |  |
| CS- | Men | -.15 | .26 | -.52 - .71 | 1.03 | 1.39 | -.44 | -.34 | -.18 | -.04 | .16 |  | .93 | .03* |  | .31 | .58 |
|  | Women | -.09 | .28 | -.76 - .41 | -.55 | -.21 | -.40 | -.27 | -.06 | .09 | .24 |  | .94 | .05* |  |  |  |
|  |  |  |  |  |  |  | Percentiles | | | | |  | Normality | |  | Homoscedasticity | |
| 1^st^ Block (Test phase) |  | Mean | SD | Range | SK | K | 10 | 25 | 50 | 75 | 90 |  | *W* | *p* |  | *F* | *p* |
| CS+ | Men | .54 | .38 | -.19 – 1.19 | -.12 | -1.23 | .04 | .26 | .54 | .87 | 1.00 |  | .96 | .24 |  | 1.18 | .28 |
|  | Women | .48 | .45 | -.35 – 1.35 | .22 | -1.17 | -.04 | .13 | .42 | .89 | 1.05 |  | .96 | .14 |  |  |  |
| CS- | Men | .05 | .39 | -1.04 - .77 | -.69 | .26 | -.40 | -.17 | .14 | .30 | .44 |  | .97 | .33 |  | .02 | .88 |
|  | Women | .14 | .39 | -.52 – 1.09 | .46 | -.32 | -.36 | -.08 | .13 | .30 | .65 |  | .96 | .14 |  |  |  |
|  |  |  |  |  |  |  | Percentiles | | | | |  | Normality | |  | Homoscedasticity | |
| 2^nd^ Block (Test phase) |  | Mean | SD | Range | SK | K | 10 | 25 | 50 | 75 | 90 |  | *W* | *p* |  | *F* | *p* |
| CS+ | Men | -.24 | .34 | -1.12 - .50 | -.08 | .30 | -.56 | -.38 | -.27 | -.07 | .21 |  | .98 | .71 |  | .44 | .51 |
|  | Women | -.26 | .30 | -.99 - .59 | .67 | 1.14 | -.54 | -.43 | -.29 | -.18 | .06 |  | .94 | .06 |  |  |  |
| CS- | Men | -.36 | .34 | -.99 - .28 | .34 | -.67 | -.67 | -.54 | -.43 | -.15 | .18 |  | .95 | .13 |  | .12 | .73 |
|  | Women | -.36 | .31 | -1.18 - .34 | .03 | -.02 | -.72 | -.55 | -.40 | -.16 | .04 |  | .98 | .85 |  |  |  |
| *Note*. The asterisk (*) indicates significance in the performed test.  SD, Standard Deviation; SK, Skewness; K, Kurtosis | | | | | | | | | | | | | | | | | |

| **Supplementary Table 2**  Pairwise comparisons between the standardized Integrated SCRs (ISCRs) generated between the first and the second block of the test phase when watching the CS- and when watching the CS+ as a function of the score in the three factors of the SPQ (X). | | | | | | | | | | |
| --- | --- | --- | --- | --- | --- | --- | --- | --- | --- | --- |
|  |  | CS- 1^st^ block vs. CS- 2^nd^ block | | | |  | CS+ 1^st^ block vs. CS+ 2^nd^ block | | | |
| Factor | X (n) | Dif [95% CI] | SE | *Ty* | *p* |  | Dif [95% CI] | SE | *Ty* | *p* |
| Cognitive-Perceptual Factor | 3 (37) | **.475 [.308, .643]** | **.082** | **5.79** | **.000** |  | **.745 [.544, .947]** | **.099** | **7.55** | **.000** |
|  | 7 (46) | **.473 [.311, .635]** | **.080** | **5.90** | **.000** |  | **.835 [.647, .1.023]** | **.093** | **8.99** | **.000** |
|  | 10 (46) | **.553 [.404, .701]** | **.073** | **7.55** | **.000** |  | **.845 [.666, 1.024]** | **.088** | **9.58** | **.000** |
|  | 13 (36) | **.381 [.194, .567]** | **.091** | **4.17** | **.000** |  | **.789 [.575, 1.004]** | **.105** | **7.52** | **.000** |
|  | 17 (25) | **.369 [.116, .621]** | **.121** | **3.05** | **.006** |  | **.706 [.469, .943]** | **.113** | **6.23** | **.000** |
| Interpersonal Factor | 5 (34) | **.369 [.207, .530]** | **.079** | **4.69** | **.000** |  | **.813 [.579, 1.047]** | **.114** | **7.12** | **.000** |
|  | 9 (50) | **.423 [.274, .572]** | **.074** | **5.74** | **.000** |  | **.805 [.631, .980]** | **.086** | **9.32** | **.000** |
|  | 13 (51) | **.461 [.312, .609]** | **.073** | **6.28** | **.000** |  | **.780 [.605, .954]** | **.086** | **9.01** | **.000** |
|  | 17 (49) | **.500 [.342, .658]** | **.078** | **6.38** | **.000** |  | **.702 [.521, .885]** | **.090** | **7.81** | **.000** |
|  | 21 (33) | **.545 [.327, .763]** | **.106** | **5.14** | **.002** |  | **.667 [.462, 1.125]** | **.100** | **6.69** | **.000** |
| Disorganized Factor | 2 (48) | **.515 [.374, .655]** | **.070** | **7.40** | **.000** |  | **.814 [.639 .989]** | **.087** | **9.40** | **.000** |
|  | 4 (56) | **.470 [.335, .605]** | **.067** | **7.02** | **.000** |  | **.795 [.634, .956]** | **.080** | **9.94** | **.000** |
|  | 6 (54) | **.459 [.302, .615]** | **.078** | **5.91** | **.000** |  | **.771 [.596, .946]** | **.087** | **8.89** | **.000** |
|  | 8 (44) | **.438 [.250, .626]** | **.093** | **4.73** | **.000** |  | **.692 [.494, .890]** | **.098** | **7.09** | **.000** |
|  | 10 (34) | **.426 [.198, .654]** | **.111** | **3.83** | **.000** |  | **.670 [.424, .915]** | **.120** | **5.60** | **.000** |
| *Note.* Pairwise comparisons that showed significant differences (*p* < .01) are boldfaced. SE, Standard Error of the difference | | | | | | | | | | |

| **Supplementary Table 3**  Pairwise comparisons between the standardized Integrated SCRs (ISCRs) generated by stimuli in the first and the second block of the test phase as a function of the total score of the SPQ (X). | | | | | | | | | | |
| --- | --- | --- | --- | --- | --- | --- | --- | --- | --- | --- |
|  |  | CS+ vs. CS- (1^st^ block of the test phase) | | | |  | CS+ vs. CS- (2^nd^ block of the test phase) | | | |
| Factor | X (n) | Dif [95% CI] | SE | *Ty* | *p* |  | Dif [95% CI] | SE | *Ty* | *p* |
| Total SPQ score | 17 (50) | **.427 [.214, .641]** | **.106** | **4.05** | **.000** |  | .024 [-.099, .147] | .061 | .40 | .691 |
|  | 22 (49) | **.402 [.197, .608]** | **.102** | **3.96** | **.000** |  | .083 [-.033, .199] | .057 | 1.45 | .155 |
|  | 27 (46) | **.420 [.222, .619]** | **.098** | **4.29** | **.000** |  | .102 [-.037, .241] | .069 | 1.49 | .145 |
|  | 32 (42) | **.399 [.181, .617]** | **.107** | **3.72** | **.001** |  | .117 [-.015, .258] | .067 | 1.81 | .080 |
|  | 37 (37) | **.399 [.157, .640]** | **.118** | **3.37** | **.002** |  | .168 [.041, .296] | .062 | 2.70 | .011 |
| *Note.* Pairwise comparisons that showed significant differences (*p* < .01) are boldfaced. SE, Standard Error of the difference | | | | | | | | | | |

| **Supplementary Table 4**  Pairwise comparisons between the standardized Integrated SCRs (ISCRs) generated between the first and the second block of the test phase when watching the CS- and when watching the CS- as a function of the total score of the SPQ (X). | | | | | | | | | | |
| --- | --- | --- | --- | --- | --- | --- | --- | --- | --- | --- |
|  |  | CS- 1^st^ block vs. CS- 2^nd^ block | | | |  | CS+ 1^st^ block vs. CS+ 2^nd^ block | | | |
| Factor | X (n) | Dif [95% CI] | SE | *Ty* | *p* |  | Dif [95% CI] | SE | *Ty* | *p* |
| Total SPQ score | 17 (50) | **.441 [.298, .585]** | **.071** | **6.23** | **.000** |  | **.844 [.673, 1.015]** | **.085** | **9.99** | **.000** |
|  | 22 (49) | **.490 [.343, .637]** | **.073** | **6.74** | **.000** |  | **.809 [.640, .978]** | **.084** | **9.68** | **.000** |
|  | 27 (46) | **.436 [.274, .597]** | **.080** | **5.46** | **.000** |  | **.754 [.560, .947]** | **.096** | **7.88** | **.000** |
|  | 32 (42) | **.435 [.259, .612]** | **.087** | **5.03** | **.000** |  | **.713 [.518, .908]** | **.096** | **7.45** | **.000** |
|  | 37 (37) | **.463 [.263, .664]** | **.098** | **4.72** | **.000** |  | **.694 [.480, .908]** | **.105** | **6.63** | **.000** |
| *Note.* Pairwise comparisons that showed significant differences (*p* < .01) are boldfaced. SE, Standard Error of the difference | | | | | | | | | | |

| **Supplementary Table 5**  Number of men and women in each group generated by the method DY depending on the score in the three factors of the SPQ as well as the total score (X) and Chi-squared statistics as a function of and sex. | | | | | |
| --- | --- | --- | --- | --- | --- |
| Factor | X (n) | n (Men) | n (Women) | *χ^2^* | *p* |
| Cognitive-Perceptual Factor | 3 (37) | 18 | 19 | 0.03 | .869 |
|  | 7 (46) | 20 | 26 | 0.78 | .376 |
|  | 10 (46) | 21 | 25 | 0.35 | .555 |
|  | 13 (36) | 16 | 20 | 0.44 | .505 |
|  | 17 (25) | 11 | 14 | 0.36 | .549 |
| Interpersonal Factor | 5 (34) | 18 | 16 | 0.12 | .732 |
|  | 9 (50) | 22 | 28 | 0.72 | .400 |
|  | 13 (51) | 24 | 27 | 0.18 | .674 |
|  | 17 (49) | 20 | 29 | 1.65 | .199 |
|  | 21 (33) | 13 | 20 | 1.48 | .223 |
| Disorganized Factor | **2 (48)** | **17** | **31** | **4.08** | **.043** |
|  | 4 (56) | 21 | 35 | 3.50 | .061 |
|  | 6 (54) | 24 | 30 | 0.67 | .414 |
|  | 8 (44) | 26 | 18 | 1.45 | .228 |
|  | 10 (34) | 22 | 12 | 2.94 | .086 |
| Total SPQ score | 17 (50) | 21 | 29 | 1.28 | .258 |
|  | 22 (49) | 22 | 27 | 0.51 | .475 |
|  | 27 (46) | 22 | 24 | 0.09 | .768 |
|  | 32 (42) | 20 | 22 | 0.10 | .758 |
|  | 37 (37) | 17 | 20 | 0.24 | .622 |
| *Note.* Significant effects (*p* < .05) are boldfaced. | | | | | |

**Supplementary Figure 1**

*Standardized integrated SCRs (ISCRs) in the learning phase as a function of stimulus and trial.*

*
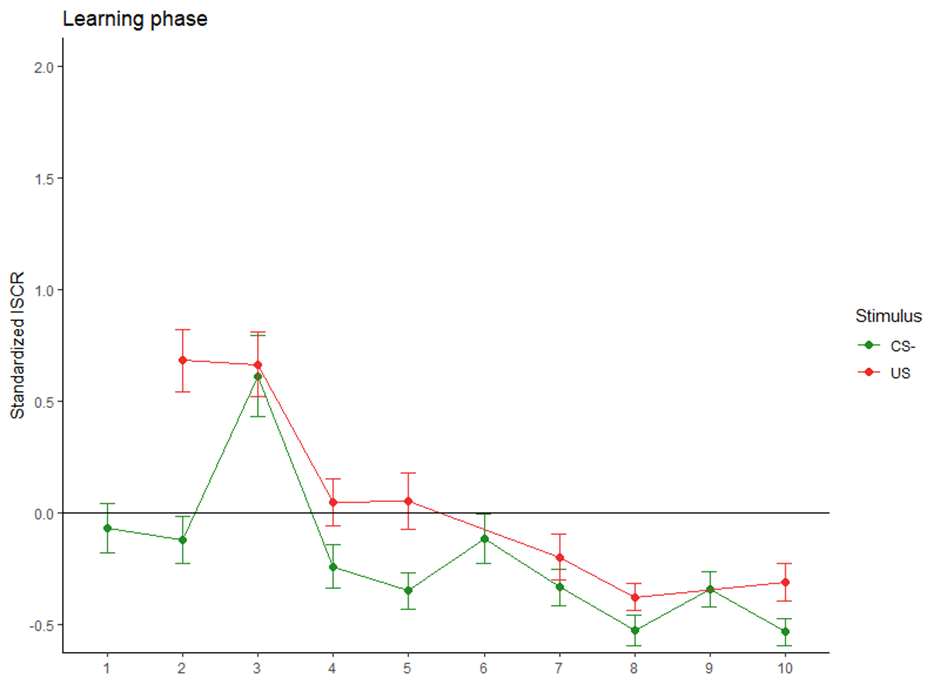
*

*Note.* Trimmed means (10%) of the standardized ISCRs of our participants as a function of stimulus and trial in the learning phase. Error bars represent the standard error of the trimmed mean.

**Supplementary Figure 2**

*Standardized integrated SCRs (ISCRs) in the test phase as a function of stimulus and trial.* *
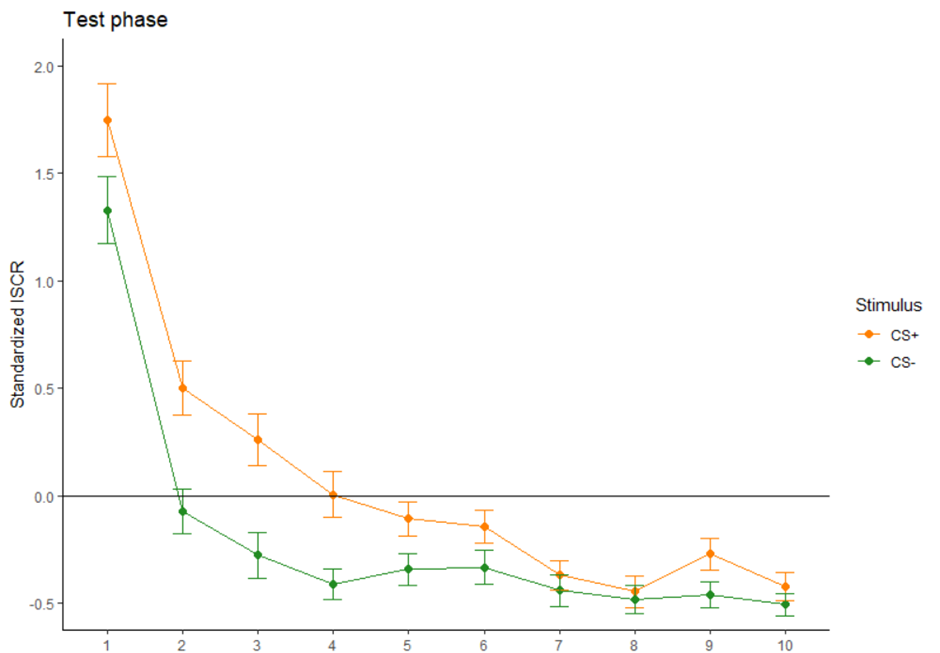
*

*Note.* Trimmed means (10%) of the standardized ISCRs of our participants as a function of stimulus and trial in the test phase. Error bars represent the standard error of the trimmed mean.

**Supplementary Figure 3**

*Standardized integrated SCRs (ISCRs) during the learning phase as a function of stimulus and the Interpersonal factor score.*


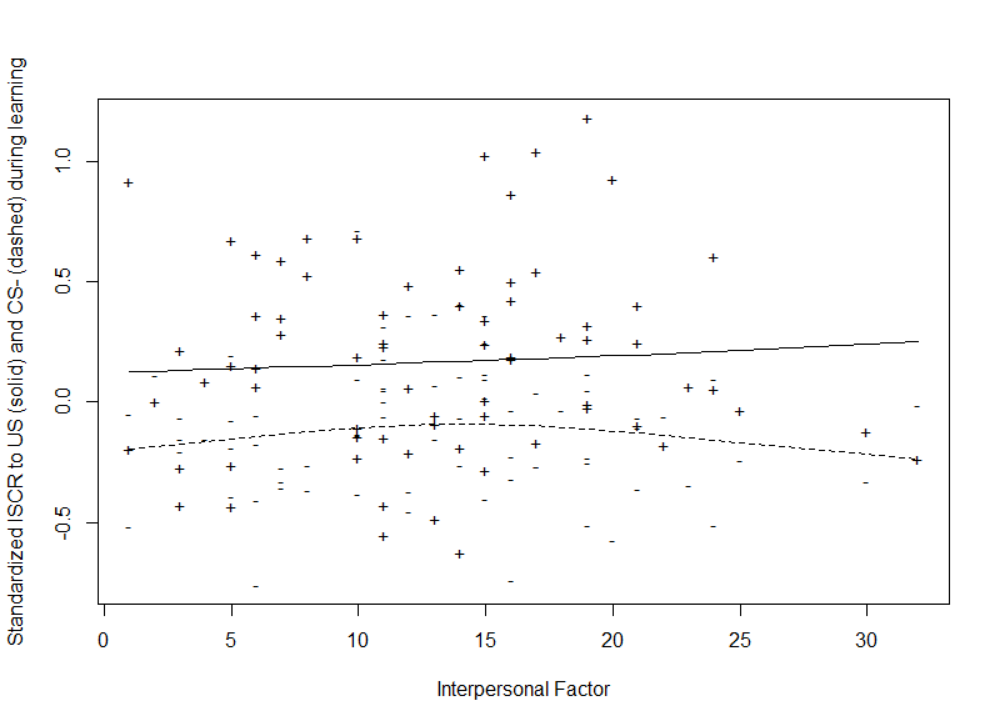


*Note.* The solid line (and the “+” symbols) represents the US and the dashed line (and the “-“ symbols) represents the CS-. Regression lines are estimated using a running interval smoother.

**Supplementary Figure 4**

*Standardized integrated SCRs (ISCRs) during the learning phase as a function of stimulus and the Disorganized factor score.*


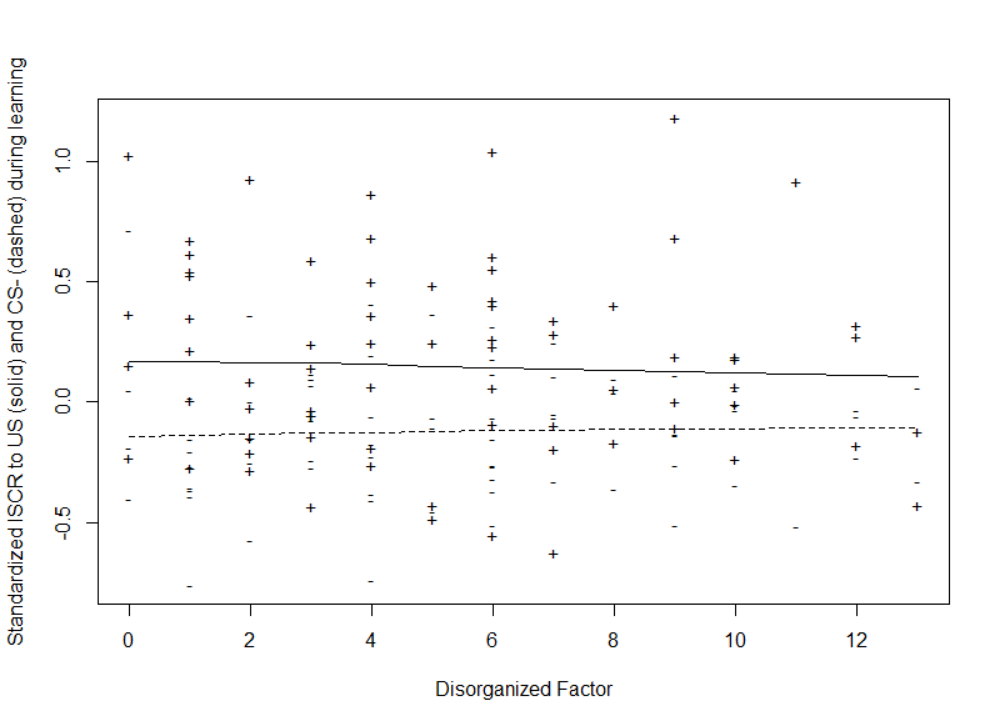


*Note.* The solid line (and the “+” symbols) represents the US and the dashed line (and the “-“ symbols) represents the CS-. Regression lines are estimated using a running interval smoother.

**Supplementary Figure 5**

*Standardized integrated SCRs (ISCRs) during the learning phase as a function of stimulus and the total score of the SPQ.*


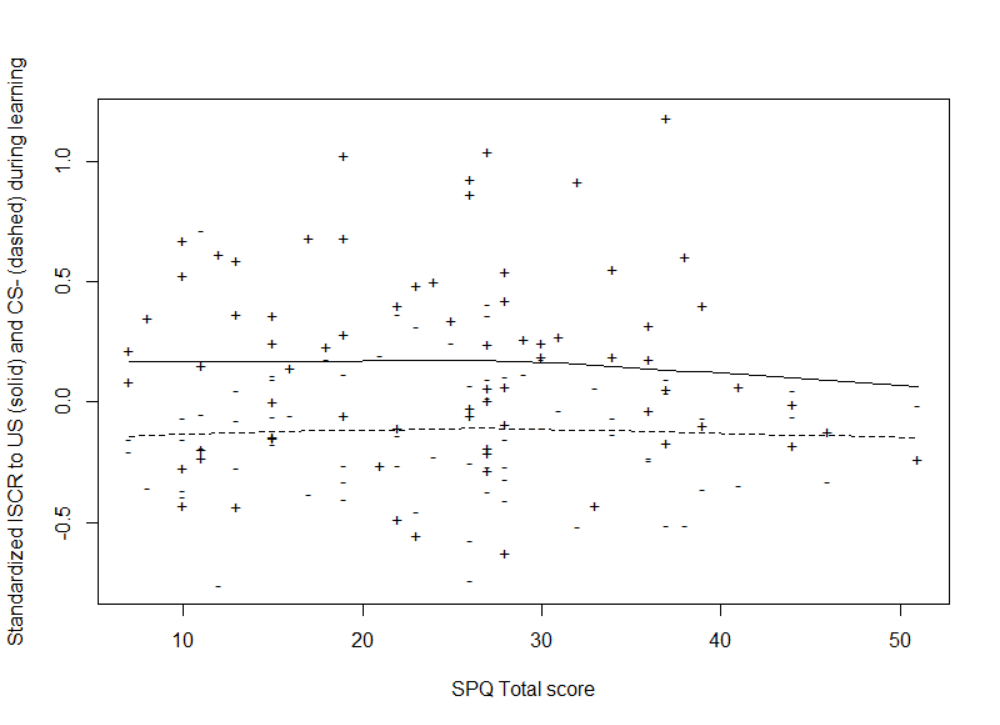


*Note.* The solid line (and the “+” symbols) represents the US and the dashed line (and the “-“ symbols) represents the CS-. Regression lines are estimated using a running interval smoother.

**Supplementary Figure 6**

*Standardized integrated SCRs (ISCRs) during the first block as a function of stimulus and the Cognitive-Perceptual factor score.*


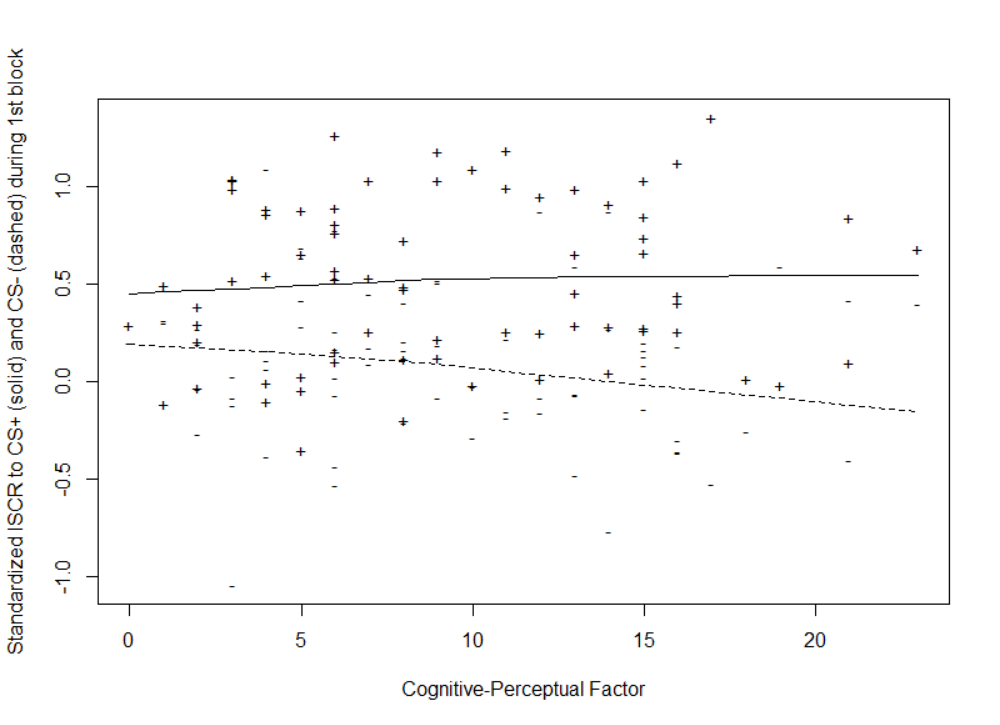


*Note.* The solid line (and the “+” symbols) represents the CS+ and the dashed line (and the “-“ symbols) represents the CS-. Regression lines are estimated using a running interval smoother.

**Supplementary Figure 7**

*Standardized integrated SCRs (ISCRs) during the first block as a function of stimulus and the Interpersonal factor score.*


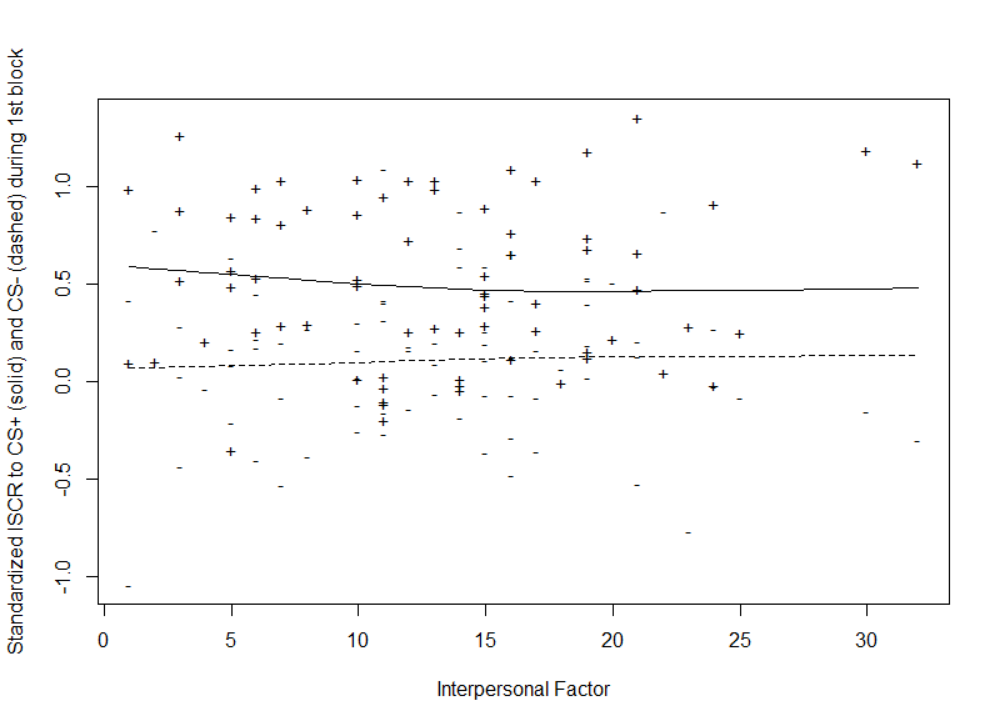


*Note.* The solid line (and the “+” symbols) represents the CS+ and the dashed line (and the “-“ symbols) represents the CS-. Regression lines are estimated using a running interval smoother.

**Supplementary Figure 8**

*Standardized integrated SCRs (ISCRs) during the first block as a function of stimulus and the Disorganized factor score.*


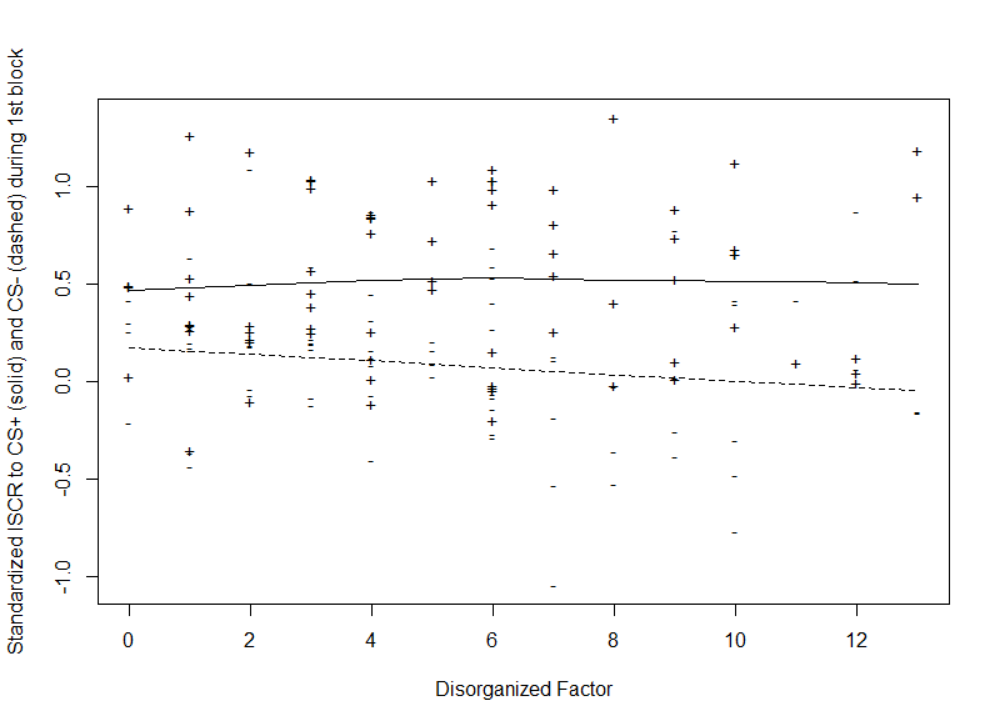


*Note.* The solid line (and the “+” symbols) represents the CS+ and the dashed line (and the “-“ symbols) represents the CS-. Regression lines are estimated using a running interval smoother.

**Supplementary Figure 9**

*Standardized integrated SCRs (ISCRs) during the first block as a function of stimulus and the total score of the SPQ.*


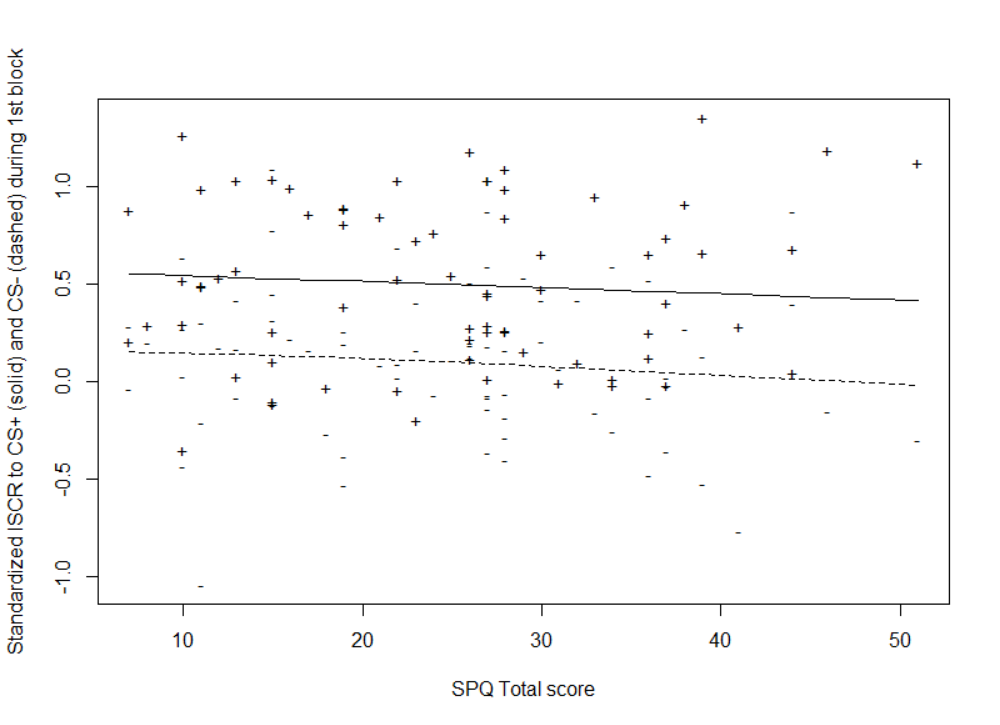


*Note.* The solid line (and the “+” symbols) represents the CS+ and the dashed line (and the “-“ symbols) represents the CS-. Regression lines are estimated using a running interval smoother.

**Supplementary Figure 10**

*Standardized integrated SCRs (ISCRs) during the second block as a function of stimulus and the Cognitive-Perceptual factor score.*


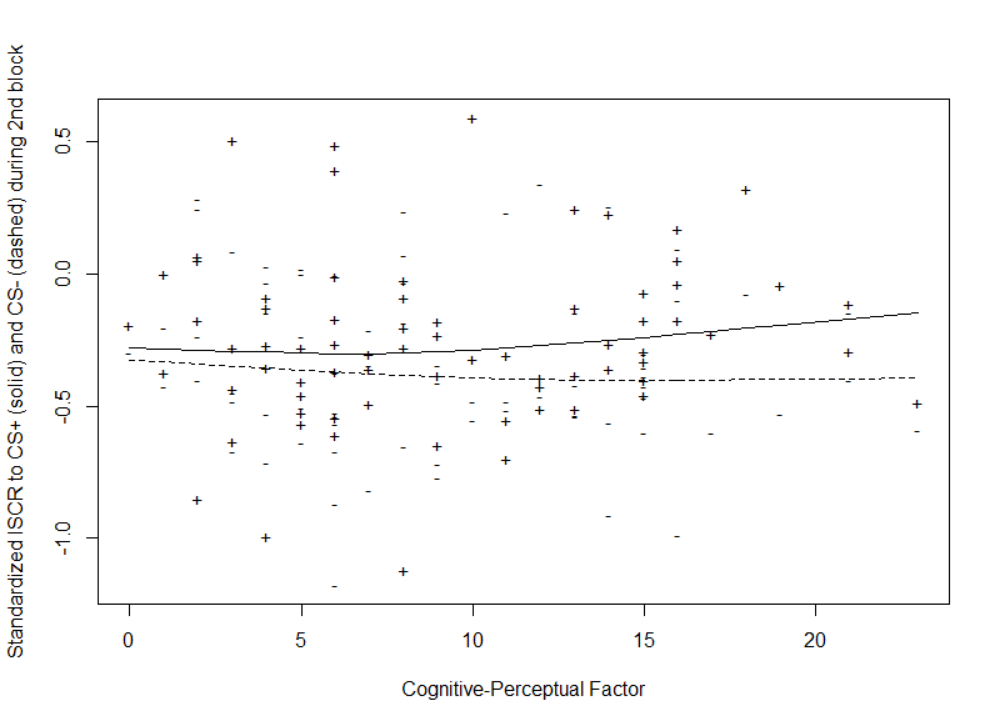


*Note.* The solid line (and the “+” symbols) represents the CS+ and the dashed line (and the “-“ symbols) represents the CS-. Regression lines are estimated using a running interval smoother.

**Supplementary Figure 11**

*Standardized integrated SCRs (ISCRs) during the second block as a function of stimulus and the Interpersonal factor score.*


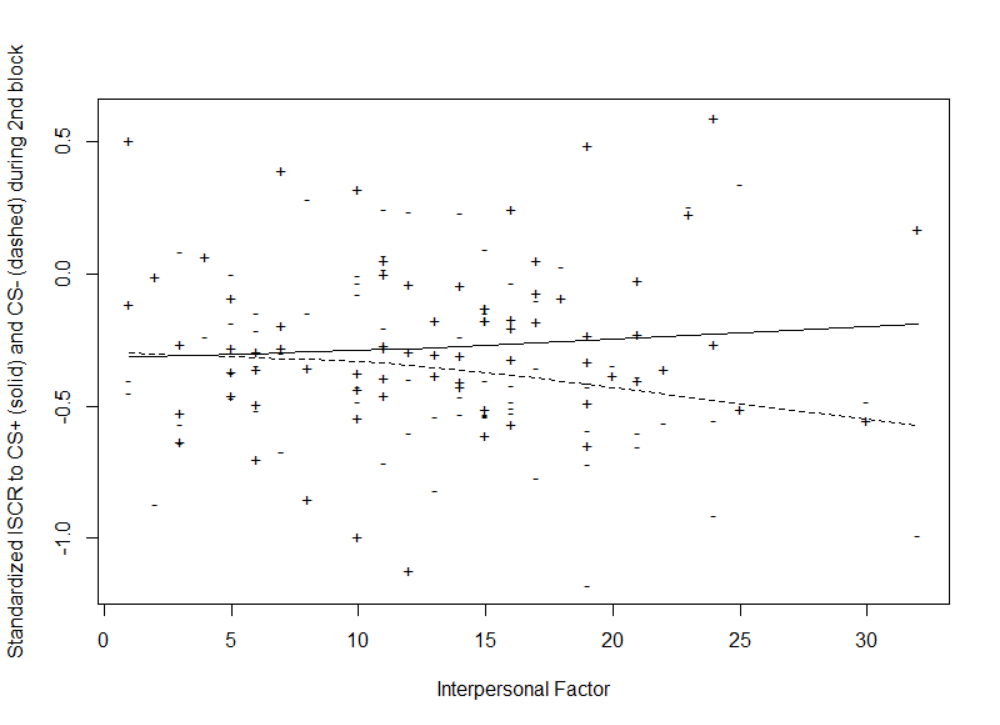


*Note.* The solid line (and the “+” symbols) represents the CS+ and the dashed line (and the “-“ symbols) represents the CS-. Regression lines are estimated using a running interval smoother.

**Supplementary Figure 12**

*Standardized integrated SCRs (ISCRs) during the second block as a function of stimulus and the total score of the SPQ.*


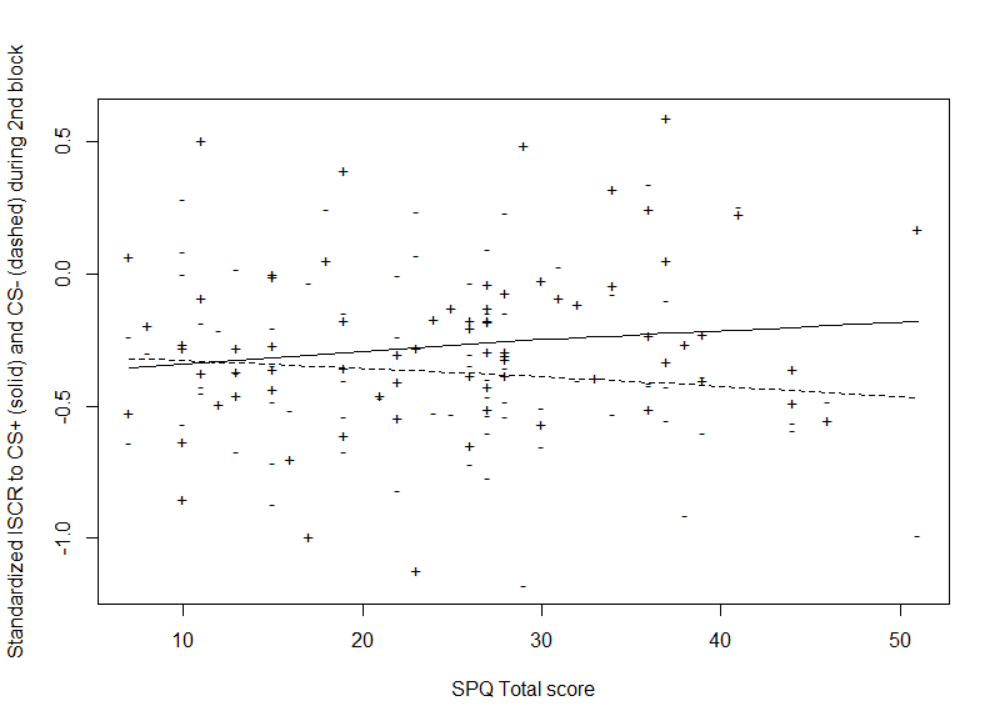


*Note.* The solid line (and the “+” symbols) represents the CS+ and the dashed line (and the “-“ symbols) represents the CS-. Regression lines are estimated using a running interval smoother.

**Supplementary Figure 13**

*Standardized integrated SCRs (ISCRs) to the CS- as a function of block and the Cognitive-Perceptual factor score.*


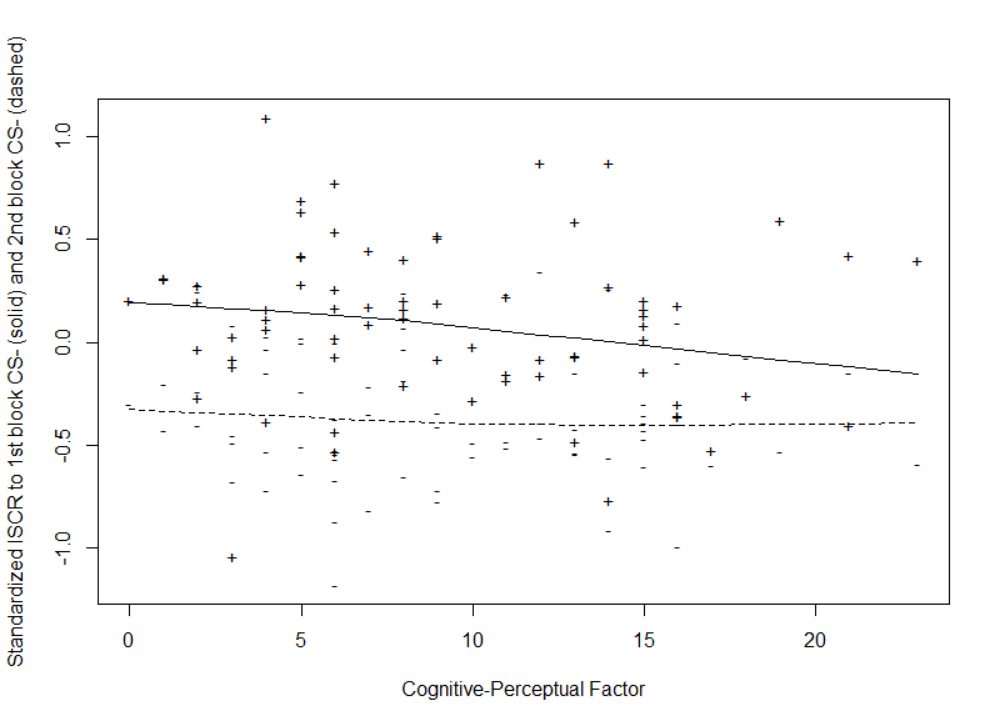


*Note.* The solid line (and the “+” symbols) represents the 1^st^ block and the dashed line (and the “-“ symbols) represents the 2^nd^ block. Regression lines are estimated using a running interval smoother.

**Supplementary Figure 14**

*Standardized integrated SCRs (ISCRs) to the CS- as a function of block and the Interpersonal factor score.*


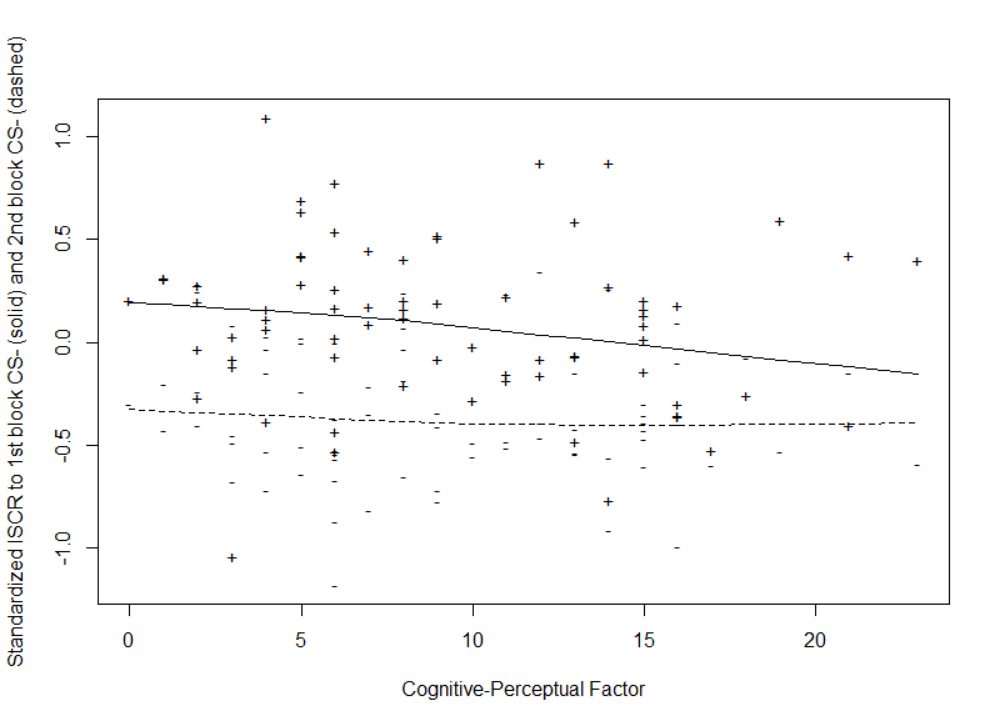


*Note.* The solid line (and the “+” symbols) represents the 1^st^ block and the dashed line (and the “-“ symbols) represents the 2^nd^ block. Regression lines are estimated using a running interval smoother.

**Supplementary Figure 15**

*Standardized integrated SCRs (ISCRs) to the CS- as a function of block and the Disorganized factor score.*


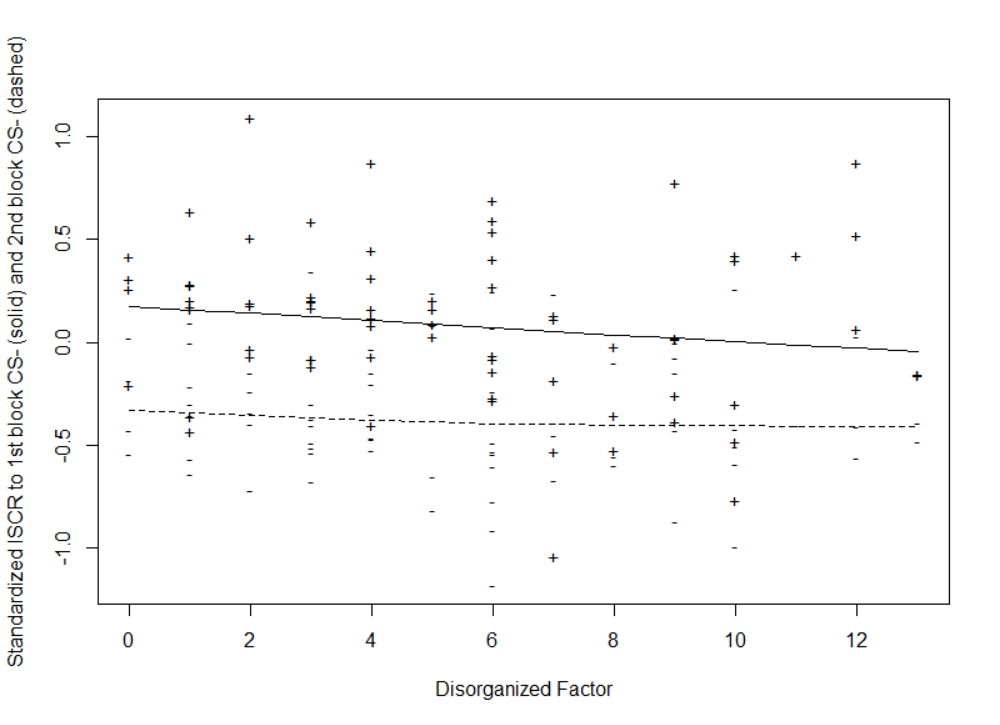


*Note.* The solid line (and the “+” symbols) represents the 1^st^ block and the dashed line (and the “-“ symbols) represents the 2^nd^ block. Regression lines are estimated using a running interval smoother.

**Supplementary Figure 16**

*Standardized integrated SCRs (ISCRs) to the CS- as a function of block and the total score of the SPQ.*


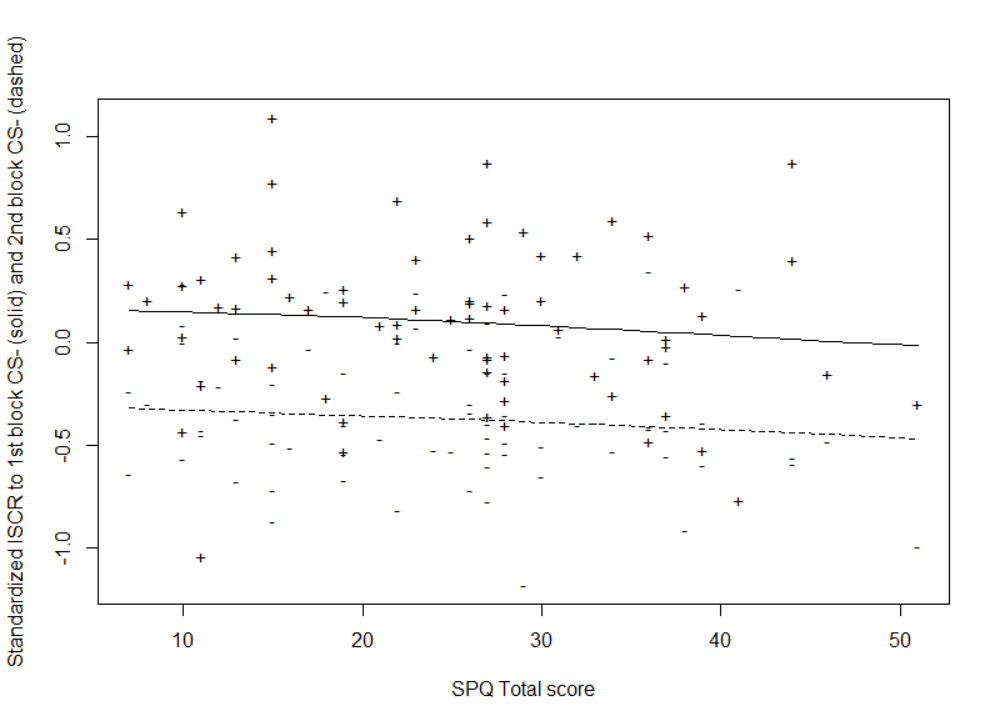


*Note.* The solid line (and the “+” symbols) represents the 1^st^ block and the dashed line (and the “-“ symbols) represents the 2^nd^ block. Regression lines are estimated using a running interval smoother.

**Supplementary Figure 17**

*Standardized integrated SCRs (ISCRs) to the CS+ as a function of block and the Cognitive-Perceptual factor score.*


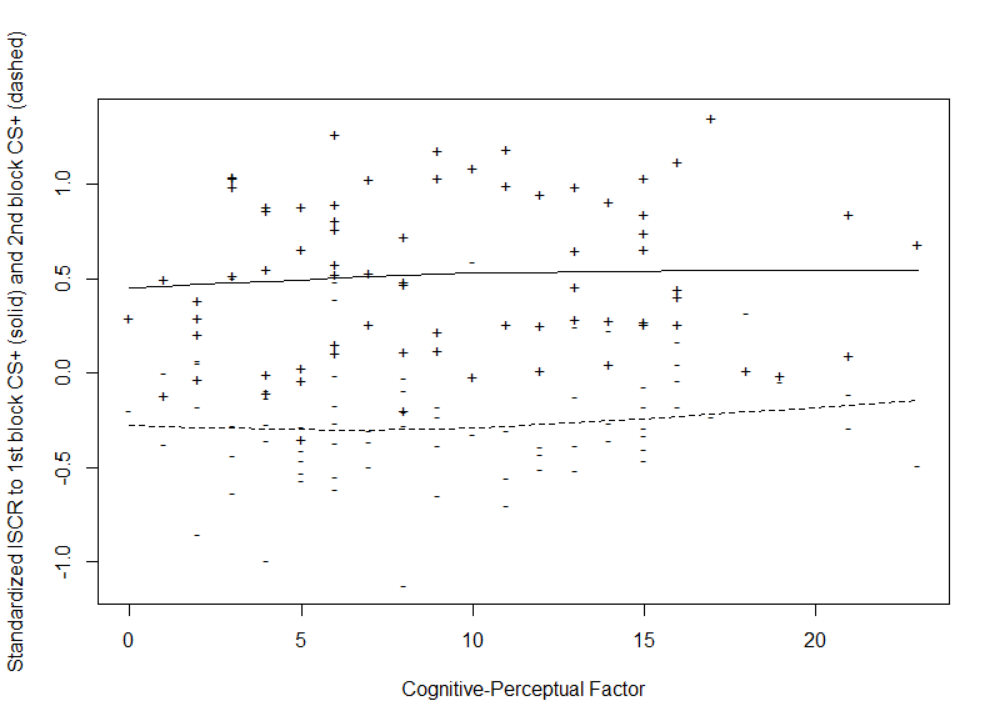


*Note.* The solid line (and the “+” symbols) represents the 1^st^ block and the dashed line (and the “-“ symbols) represents the 2^nd^ block. Regression lines are estimated using a running interval smoother.

**Supplementary Figure 18**

*Standardized integrated SCRs (ISCRs) to the CS+ as a function of block and the Interpersonal factor score.*


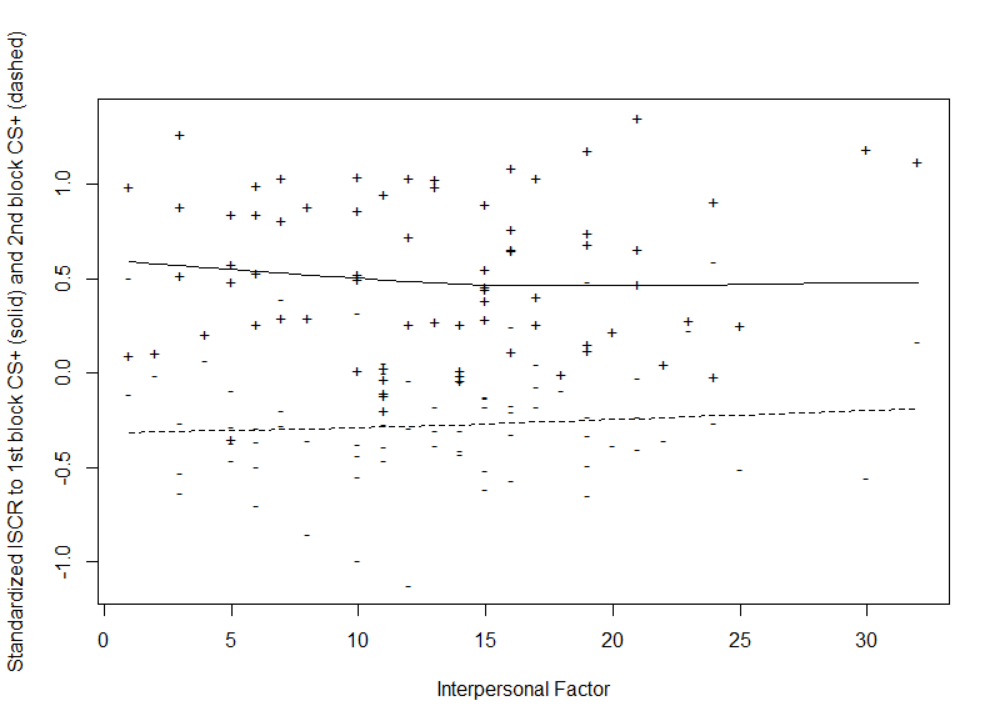


*Note.* The solid line (and the “+” symbols) represents the 1^st^ block and the dashed line (and the “-“ symbols) represents the 2^nd^ block. Regression lines are estimated using a running interval smoother.

**Supplementary Figure 19**

*Standardized integrated SCRs (ISCRs) to the CS+ as a function of block and the Disorganized factor score.*


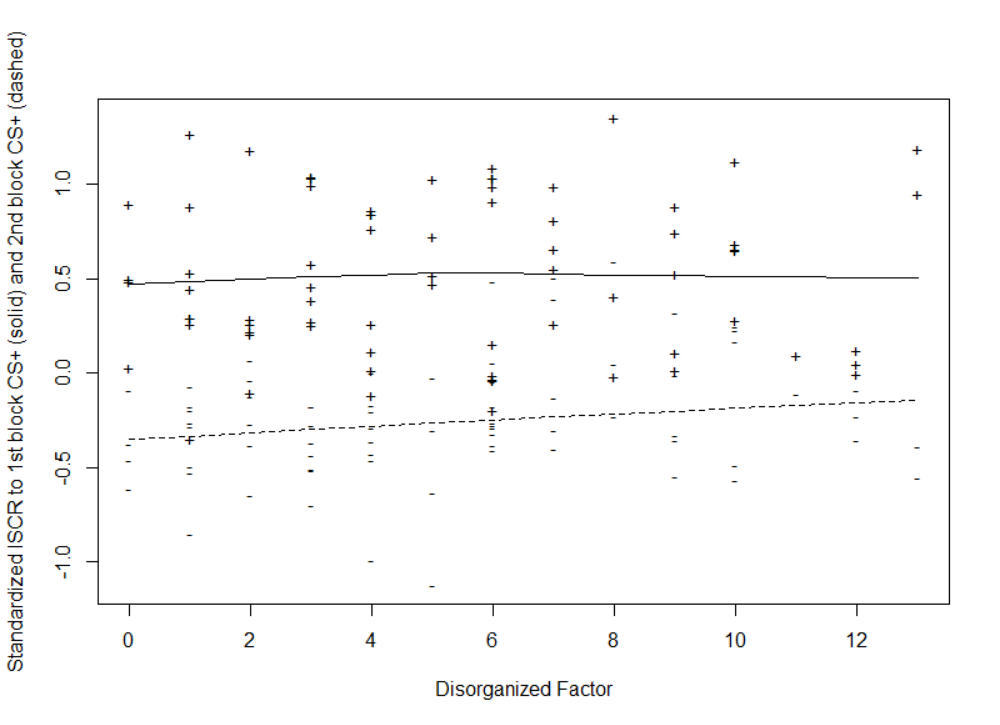


*Note.* The solid line (and the “+” symbols) represents the 1^st^ block and the dashed line (and the “-“ symbols) represents the 2^nd^ block. Regression lines are estimated using a running interval smoother.

**Supplementary Figure 19**

*Standardized integrated SCRs (ISCRs) to the CS+ as a function of block and the total score of the SPQ.*


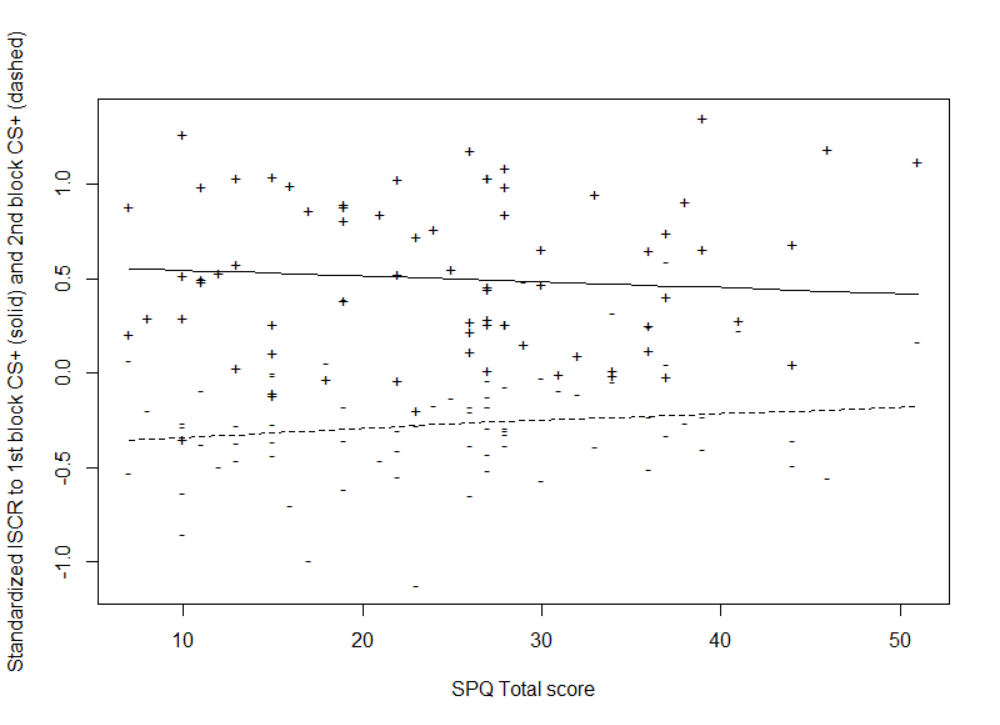


*Note.* The solid line (and the “+” symbols) represents the 1^st^ block and the dashed line (and the “-“ symbols) represents the 2^nd^ block. Regression lines are estimated using a running interval smoother.
